# Exercise programmes incorporating proximal kinetic-chain components in flexible flatfoot: a systematic review and meta-analysis of foot posture, neuromuscular, and dynamic outcomes

**DOI:** 10.3389/fphys.2026.1959545

**Published:** 2026-10-12

**Authors:** Zheyu Xiong, Jiguo Liang, Guanghua Li

**Affiliations:** 1 School of Sports and Health Engineering, Hebei University of Engineering, Handan, Hebei, China; 2 School of Physical Education and Health Science, Guangxi Minzu University, Nanning, Guangxi, China

**Keywords:** exercise therapy, flexible flatfoot, foot posture, neuromuscular adaptation, proximal kinetic chain

## Abstract

**Purpose:**

To evaluate the effects of exercise programmes incorporating proximal kinetic-chain components versus local or basic foot–ankle exercise in individuals with study-defined flexible flatfoot or low-arch/pronated-foot phenotypes.

**Methods:**

PubMed, Web of Science, Scopus, and SPORTDiscus were searched from inception through 19 July 2026. Randomised controlled trials comparing exercise programmes incorporating proximal kinetic-chain components with local or basic foot–ankle exercise programmes were included. Random-effects meta-analyses used restricted maximum likelihood estimation. Risk of bias was assessed with RoB 2 and certainty of evidence with GRADE.

**Results:**

Fourteen randomised trials reported in 15 publications were included; 13 trials (14 reports) contributed to at least one meta-analysis. Programmes containing proximal or kinetic-chain components were associated with lower navicular-drop values (11 trials, 410 participants; mean difference −2.34 mm, 95% CI −3.30 to −1.37; I²=84.6%; low-certainty evidence). The Foot Posture Index estimate was uncertain (4 trials, 157 participants; mean difference −1.67 points, 95% CI −3.36 to 0.01; I²=89.1%; very-low-certainty evidence). The exploratory Plantar Arch Index and all three exploratory Y-Balance estimates had confidence intervals including the null and very-low-certainty evidence. Evidence for muscle, plantar-pressure, gait, kinematic, patient-reported, adherence, and adverse-event outcomes was limited and mainly derived from individual trials.

**Conclusion:**

Programmes incorporating proximal kinetic-chain components may reduce short-term navicular drop compared with local or basic foot–ankle exercise programmes, but substantial heterogeneity limits confidence in the magnitude of this response; effects on FPI-6 remain uncertain. Evidence for dynamic, functional, and patient-reported outcomes remains insufficient.

**Systematic review registration:**

identifier CRD420261455079.

## 1 Introduction

Flexible flatfoot is a load-dependent phenotype in which the medial longitudinal arch remains mobile and partly or fully reconstitutes during non-weight-bearing, tiptoe, or corrective manoeuvres, distinguishing it from rigid or non-reducible flatfoot (Mosca, 2010). Pronated foot posture describes static alignment and does not, by itself, establish arch flexibility. These overlapping constructs should therefore not be treated as identical. A flexible low arch may be an asymptomatic morphological variant rather than a disorder requiring correction (Halabchi et al., 2013). Its behaviour reflects the interaction of passive structures, intrinsic and extrinsic foot muscles, sensory feedback, and multisegment coordination during weight-bearing, as described by the foot-core framework (McKeon et al., 2015).

Local foot and foot–ankle exercise provides a direct stimulus to the distal neuromuscular system. Short-foot exercise, intrinsic-foot-muscle training, toe-control tasks, towel curls, and selective foot–ankle strengthening are intended to enhance active arch support, local force production, and sensorimotor control (McKeon et al., 2015; Huang et al., 2022; Wei et al., 2022; Jaffri et al., 2023). In practice and research, however, these exercises are often combined with hip or gluteal strengthening, core-stability exercise, closed-chain lower-extremity tasks, Pilates, balance training, or gait and movement-control training. These proximal components provide a different training stimulus by targeting hip and lumbopelvic capacity, lower-limb alignment, force attenuation, and multisegment coordination during weight-bearing tasks. Conservative care also includes foot orthoses and customised insoles; studies of these approaches provide complementary clinical context but address a different intervention question (Öztürk and Kurul, 2024).

A proximal-to-distal adaptation pathway is biomechanically plausible because hip and pelvic control, femoral and tibial rotation, knee alignment, foot pronation, and plantar loading are mechanically linked during weight-bearing activities (Buldt et al., 2013; Resende et al., 2015; Hornestam et al., 2021). Exercise-induced changes in proximal neuromuscular capacity could therefore influence distal foot–ankle behaviour through altered segmental coordination and load transfer (**Figure 1**). Nevertheless, biomechanical plausibility does not demonstrate that proximal training independently modifies foot function, and evidence derived from acute or associative studies cannot establish longitudinal adaptation.

**Figure 1:**
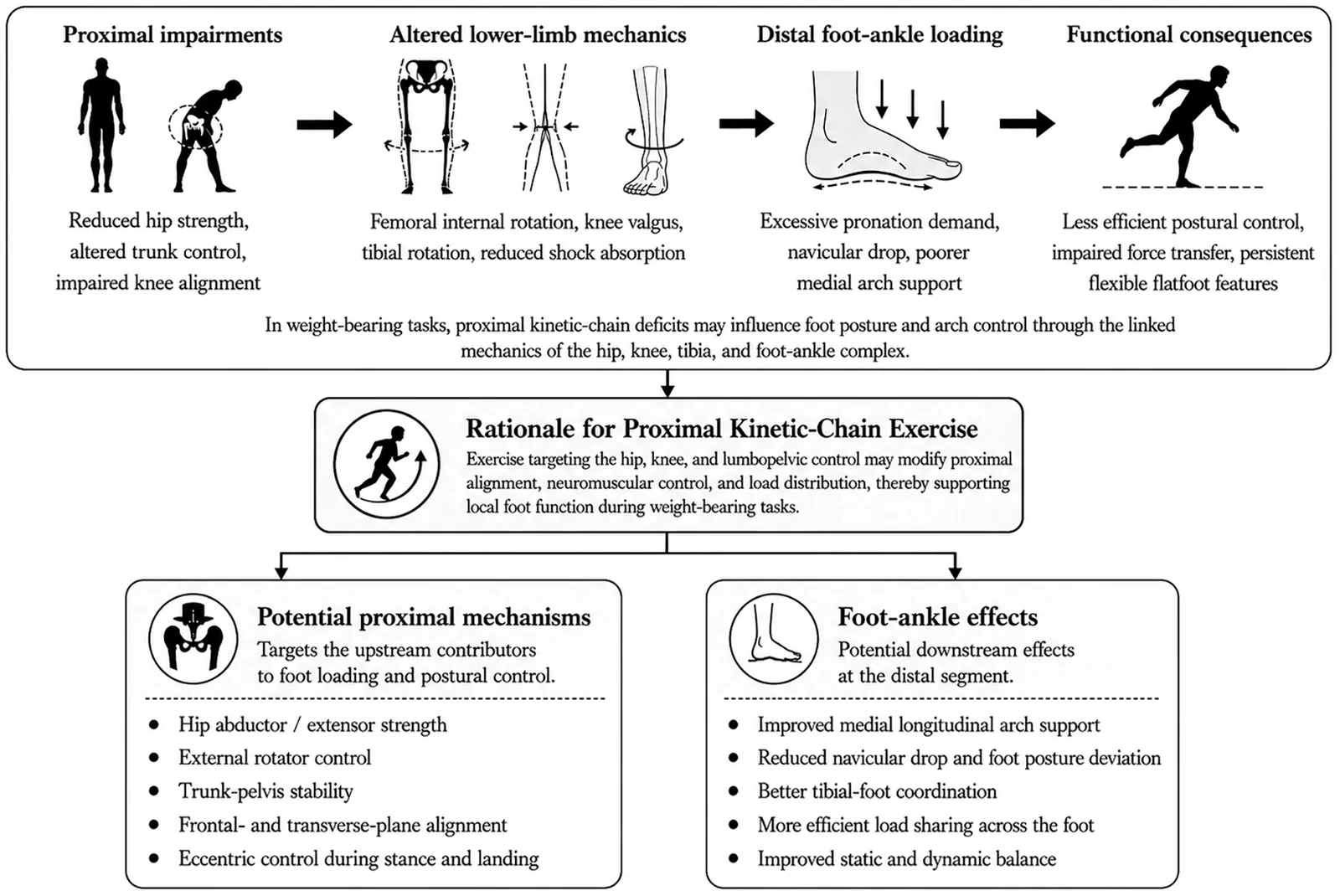
Proposed proximal-to-distal biomechanical framework in flexible flatfoot.

The interpretation of exercise responses also depends on the outcome construct. Navicular drop, the Foot Posture Index-6, arch angles, and footprint-derived indices quantify related but non-equivalent aspects of static foot posture (Redmond et al., 2006; Huang et al., 2022; Wei et al., 2022; Jaffri et al., 2023). Muscle strength, proprioception, electromyography, plantar-pressure distribution, gait, lower-limb kinematics, and postural-control tests represent distinct neuromuscular, mechanical, and functional domains. A change in static foot posture may indicate a distal response to training, but it does not by itself demonstrate broader neuromuscular adaptation or transfer to dynamic function. Establishing such transfer requires concurrent assessment of proximal capacity, distal loading, segmental mechanics, and task performance.

Previous reviews have synthesised local foot exercise (Huang et al., 2022; Wei et al., 2022; Jaffri et al., 2023) or broader exercise approaches (Jia et al., 2024; Cömert and Gruber, 2025). A recent review of hip-focused exercise concentrated on medial longitudinal arch height and also described balance and foot-posture findings (Mutlu and Yağcı, 2026). The present review complements that work by specifically comparing programmes incorporating proximal kinetic-chain components with local active foot–ankle exercise and by synthesising neuromuscular, plantar-pressure, gait, balance, and patient-reported outcomes alongside static foot posture. A comparison in which groups differ only by a proximal component can more directly inform component attribution (Engkananuwat and Kanlayanaphotporn, 2023; Zarali et al., 2024), whereas differences in local exercise, dose, progression, supervision, or co-interventions primarily estimate an overall programme effect (Brijwasi and Borkar, 2023). Accordingly, this review evaluated these programme-level comparisons and examined whether static foot-posture responses were accompanied by broader neuromuscular, biomechanical, or dynamic functional changes.

## 2 Methods

### 2.1 Protocol registration and reporting

The protocol for this systematic review and meta-analysis was prospectively registered in PROSPERO (CRD420261455079). The review followed the PRISMA 2020 statement (Page et al., 2021), and methodological procedures were informed by the Cochrane Handbook for Systematic Reviews of Interventions (Higgins et al., 2024).

### 2.2 Information sources and search strategy

PubMed, Web of Science Core Collection, Scopus, and SPORTDiscus with Full Text via EBSCOhost were searched from inception through the final platform-specific searches conducted on 18–19 July 2026. Strategies combined controlled vocabulary, where available, with free-text terms for flexible flatfoot, low medial longitudinal arch or excessive pronation; local foot or foot–ankle exercise; and proximal, hip, core, or kinetic-chain exercise. Boolean operators, truncation, proximity operators, and field restrictions were adapted to each database. Complete database- and platform-specific strategies are provided in **Supplementary Material 1** (Rethlefsen et al., 2021).

Supplementary searching included Europe PMC, ClinicalTrials.gov, backward screening of reference lists from eligible reports and relevant reviews, and forward citation searching. Registry records and grey-literature reports were assessed against the same eligibility criteria as journal articles. No restrictions were imposed on language, publication date, or publication status. Potentially eligible non-English reports were translated when necessary and feasible.

### 2.3 Eligibility criteria

Eligible participants of any age or sex, with or without symptoms, had flexible, functional, or mobile flatfoot; a flexible low medial longitudinal arch; or excessive or pronated foot posture meeting a prespecified clinical threshold. Flexibility could be confirmed by the Jack test, tiptoe or heel-rise testing, restoration of the arch in non-weight-bearing, or an equivalent examination. Studies using navicular drop, the Foot Posture Index, footprint indices, or comparable posture thresholds remained eligible. Rigid or non-reducible flatfoot and relevant structural, congenital, neurological, traumatic, inflammatory, or postoperative conditions were excluded unless eligible data were separable. Diagnostic evidence was additionally classified as an explicitly reported flexibility test or a posture/navicular-drop threshold without a separately reported flexibility test.

Eligible interventions were repeated exercise programmes incorporating proximal kinetic-chain components, including hip, core, gluteal, closed-chain lower-extremity, Pilates, functional gait, or other kinetic-chain elements. Eligible comparisons evaluated these programmes against local foot, intrinsic-foot-muscle, short-foot, toe-spread, towel-curl, foot–ankle, or basic active ankle exercise programmes. Local exercise content, total exercise volume, progression, supervision, session duration, and other active components could differ across trials and were extracted to inform attribution and the interpretation of clinical heterogeneity. The primary synthesis compared exercise programmes incorporating proximal kinetic-chain components with local or basic foot–ankle exercise programmes. Comparator similarity and component matching were examined to inform interpretation, but pooled estimates were regarded primarily as programme-level effects because intervention content, exercise dose, and co-interventions frequently differed between groups. Single-session acute experiments, passive-only programmes, and programmes without an eligible proximal component or active exercise comparator were excluded.

Eligible outcomes included static foot posture, balance and postural control, strength, plantar pressure, gait or kinematics, electromyography, symptoms, function, and quality of life. Navicular drop, converted to millimetres, was the principal static foot-posture outcome. The intervention-end assessment was used for the primary analysis; later follow-up assessments were synthesised separately when appropriate.

Only randomised controlled trials with a concurrent eligible comparator were included. Uncontrolled pre-post studies, quasi-randomised or non-randomised studies, cross-sectional studies, case reports or case series, reviews, protocols without completed data, conference abstracts lacking sufficient full-text information, and animal or cadaveric studies were excluded.

For each specific comparison contributing to an outcome synthesis, comparisons were classified as component-matched (CM), partially matched (PM), or unmatched multicomponent (UM). CM indicated substantially similar background local exercise content. PM indicated differences in training task, progression, supervision or ancillary exercise exposure, or insufficient reporting to establish local-dose matching. UM indicated additional distinct local treatment components beyond proximal exercise.

### 2.4 Study selection and data extraction

After deduplication, two reviewers independently screened titles and abstracts and independently assessed potentially eligible full texts. Disagreements were resolved by discussion or, when unresolved, adjudication by a third reviewer. Reasons for full-text exclusion were recorded, and multiple reports from the same trial were linked at study level. The selection process was documented in a PRISMA flow diagram.

A standardised extraction form was developed prospectively. Two reviewers independently completed data extraction against the original reports; disagreements were resolved by discussion or, when unresolved, adjudication by a third reviewer. Extracted information covered study design and setting; participant and diagnostic characteristics; numbers randomised and analysed; intervention and comparator components; exercise frequency, duration, intensity, volume, progression, supervision, and adherence; co-interventions and adverse events; and outcome definitions, measurement instruments, analysed foot or limb, and time points. Arm-level data included sample size, mean, SD, median, interquartile range, standard error, confidence interval, and other statistics required for effect-size calculation. Randomisation and analysis units, shared comparators, bilateral-foot outcomes, and potentially overlapping reports were also recorded. Intervention and comparator content, total exercise dose, supervision, progression, and co-interventions were extracted to support interpretation of between-study clinical heterogeneity.

Numerical data were obtained preferentially from tables, followed by numerical text and **Supplementary Materials**. Original values were stored separately from calculated or converted data. Values with irreconcilable inconsistencies or undefined meaning were excluded from quantitative synthesis.

### 2.5 Risk of bias, reporting bias, and certainty of evidence

Two reviewers independently assessed risk of bias, with disagreements resolved by discussion or adjudication by a third reviewer. Randomised trials were assessed using Cochrane Risk of Bias 2 (RoB 2) (Sterne et al., 2019) across five domains: bias arising from the randomisation process; bias due to deviations from intended interventions; bias due to missing outcome data; bias in measurement of the outcome; and bias in selection of the reported result. Each domain and the overall assessment were classified as low risk, some concerns, or high risk, and assessments were outcome-specific when appropriate. Traffic-light and summary plots were produced using the robvis package (McGuinness and Higgins, 2021). Summed methodological quality scores were not calculated or used.

Selective reporting within individual trials was assessed under the RoB 2 domain concerning selection of the reported result (Sterne et al., 2019). For outcome syntheses containing at least 10 independent studies, funnel plots were visually inspected, and Egger’s regression test was used to assess small-study effects (Egger et al., 1997; Higgins et al., 2024). Formal funnel-plot asymmetry tests were not undertaken when fewer than 10 independent studies contributed, and publication bias was instead considered qualitatively in the GRADE assessment (Higgins et al., 2024). A non-significant Egger test was not interpreted as evidence that publication bias was absent.

Two reviewers independently assessed certainty of evidence using GRADE (Guyatt et al., 2008) for each principal quantitative outcome; disagreements were resolved by discussion or a third reviewer. Randomised evidence initially began at high certainty and could be downgraded for risk of bias, inconsistency, indirectness, imprecision, and publication bias. Certainty was classified as high, moderate, low, or very low. Summary of Findings tables were produced using GRADEpro GDT. Inconsistency considered differences in effect magnitude and interval overlap alongside clinical diversity, rather than I² alone. Indirectness was judged against the eligible populations, whole-programme comparisons, measured outcomes, and intervention-end time points; it did not address isolated proximal effects or unmeasured patient-important benefit. Detailed rationales are provided in **Supplementary Material S4**.

### 2.6 Data synthesis and statistical analysis

Final post-intervention values were used for the primary analysis. Final-value and change-score estimates from the same comparison were not entered together; change scores were reserved for sensitivity analysis. Mean differences (MDs) were pooled when the same outcome was reported on the same scale, and Hedges g was used when the same construct was measured with different scales or units (Higgins et al., 2024). Navicular drop was converted to millimetres; the Foot Posture Index and comparable arch indices were retained on their original scales. Distinct clinical or biomechanical constructs were not pooled solely because a standardised effect could be calculated. Effects were calculated as intervention minus comparator, and forest plots explicitly labelled the direction favouring each group.

Missing SDs were derived, when mathematically valid, from standard errors, 95% confidence intervals, exact p values, or test statistics. The standard error of measurement was not treated as a between-participant SD. When only medians and interquartile ranges were reported, means were estimated using the Luo method (Luo et al., 2018) and SDs using the Wan method (Wan et al., 2014).

When a change-score SD was not reported but baseline and follow-up SDs were available, it was estimated for change-score sensitivity analyses using Equation 1, according to Cochrane guidance (Higgins et al., 2024):

(1) $$SD_{\text{change}}=\sqrt{SD_{\text{baseline}}2^{+}SD_{\text{follow}-\text{up}}2^{-}2\times Corr\times SD_{\text{baseline}}\times SD_{\text{follow}-\text{up}}}$$

Corr represents the correlation between baseline and follow-up measurements. Change-score sensitivity analyses were conducted using assumed pre–post correlations of 0.30 and 0.70.

The participant was the preferred independent unit. For bilateral outcomes, the study-specified, dominant, target, or affected foot was used when available; otherwise, right-foot data were used. Both feet from one participant were not treated as independent. When foot-level analyses ignored within-participant clustering, effective sample sizes were adjusted using a design effect calculated with Equation 2, in accordance with Cochrane unit-of-analysis guidance (Higgins et al., 2024):

(2) DE=1+(m−1)×ICC

DE is the design effect, m is the number of feet contributed by each participant, and ICC is the intraparticipant correlation between feet. The adjusted effective sample size was calculated using Equation 3:

(3) $$n_{\text{eff}}=\frac{n_{\text{obs}}}{DE}$$

n_eff is the adjusted effective sample size and n_obs is the reported number of foot-level observations. Reported means and SDs were retained unless participant-level estimates were available. For Engkananuwat (Engkananuwat and Kanlayanaphotporn, 2023), each group contributed 52 feet from 26 participants. A conservative ICC of 1 was assumed (m=2; DE = 2), yielding an effective sample size of 26 per group while retaining the published foot-level means and SDs.

When two eligible intervention arms shared one comparator in the same outcome synthesis, the intervention arms were combined, and the comparator was included once. Reports were linked at study level and were not treated as independent within the same outcome synthesis unless sample independence was established.

Quantitative pooling required at least two clinically and methodologically comparable studies. Inverse-variance random-effects models were used, with between-study variance estimated using restricted maximum likelihood in all random-effects analyses (Higgins et al., 2024). Statistical heterogeneity was reported using model-based I² (Higgins et al., 2003), τ², and Cochran’s Q. Knapp–Hartung-adjusted confidence intervals were used when at least three studies contributed to a synthesis (k ≥ 3) (IntHout et al., 2014). For syntheses containing only two studies (k = 2), a random-effects model with restricted maximum-likelihood estimation and conventional z-based Wald-type 95% confidence intervals was used; these analyses were considered exploratory. Pooled MDs or Hedges g values were reported with 95% confidence intervals. Analyses were conducted in R version 4.6.1 using metafor version 5.0–1 (Viechtbauer, 2010).

Forest plots displayed study-level effect estimates with 95% confidence intervals, the effect metric (MD or Hedges g), the pooled random-effects estimate, and the direction favouring each group. I² and τ² were displayed where applicable.

When quantitative pooling was inappropriate, findings were synthesised narratively by outcome construct, measurement task, and time point, following SWiM guidance (Campbell et al., 2020).

### 2.7 Sensitivity analysis

Change-score sensitivity analyses used assumed pre–post correlations of 0.30 and 0.70. Targeted study-exclusion analyses assessed the influence of individual studies and bilateral-foot data handling. Exploratory analyses assessed the influence of programme matching by separately excluding PM, UM, or CM comparisons and by restricting the synthesis to CM comparisons. Results from a single remaining trial were not pooled.

## 3 Results

### 3.1 Study selection

The database and register searches identified 6,543 records (PubMed, n=1,339; Web of Science, n=1,622; Scopus, n=2,760; SPORTDiscus, n=822). After 2,702 duplicate records were removed through identifier/title matching, 3,841 records were screened and 3,810 were excluded. Full texts were sought for 31 reports, of which four could not be retrieved. Twenty-seven reports were assessed for eligibility, and 13 were excluded because there was no eligible or verifiable comparator (n=2), a non-exercise co-intervention was unequal between groups (n=2), the active comparison was outside the review scope (n=6), the report was a corrigendum without independent eligible outcome data (n=1), or the intervention was ineligible (n=2). Fourteen reports were included through databases and registers. Report availability and documented retrieval attempts are detailed in **Supplementary Material S5**.

A further 11 reports were identified through other methods, all of which were retrieved and assessed for eligibility. Ten were excluded because of an ineligible population (n=3), intervention or comparator (n=4), study design (n=2), or outcome (n=1), leaving one report included from other methods. Overall, 15 reports represented 14 independent RCTs, with the two Zarali reports (Zarali and Raeisi, 2023; Zarali et al., 2024) describing the same trial. Thirteen RCTs (14 reports) contributed data to at least one quantitative synthesis. One study was retained for qualitative synthesis only because it reported no outcome sufficiently comparable for meta-analysis. The study-selection process is shown in **Figure 2**.

**Figure 2:**
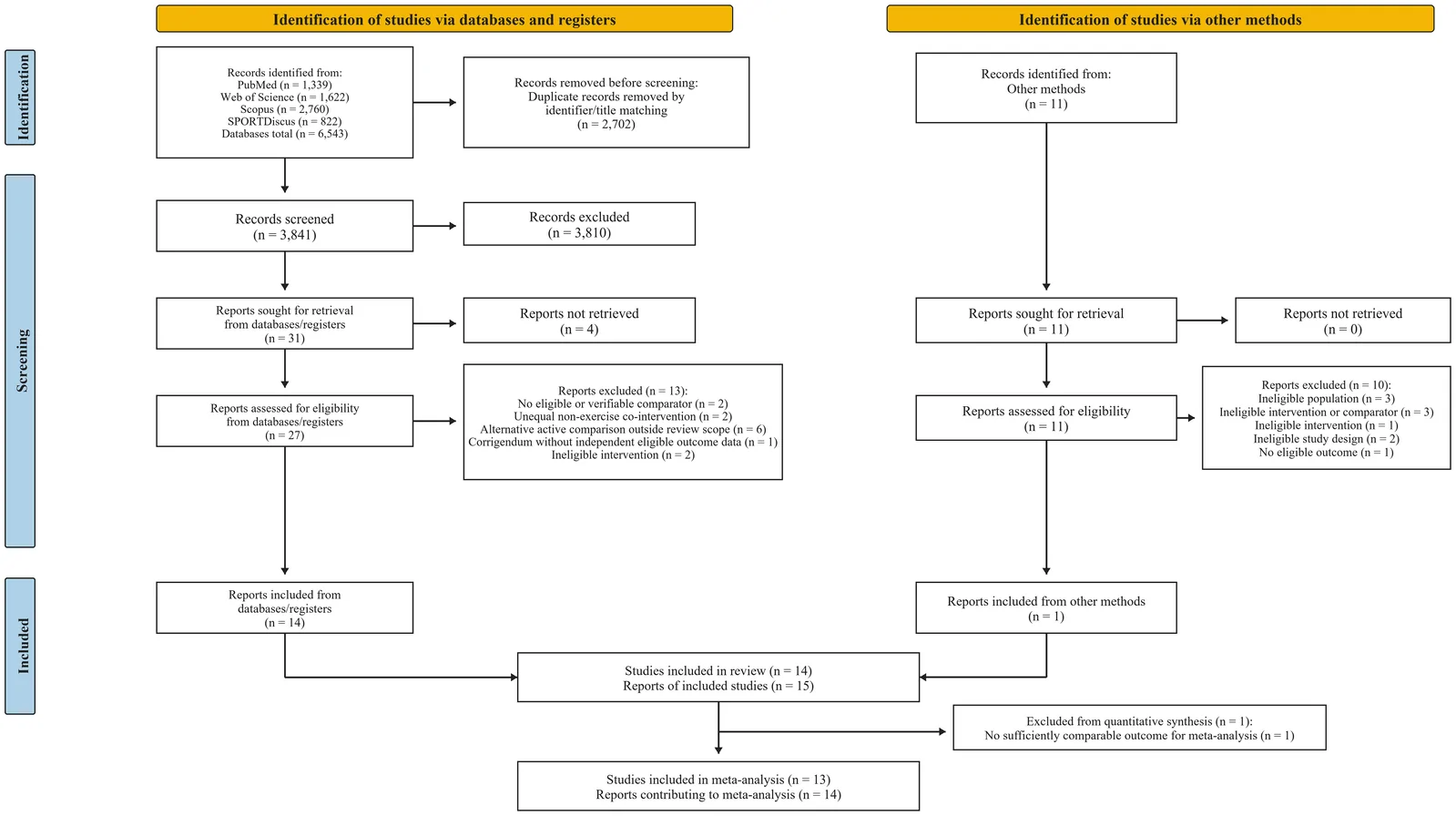
PRISMA flow diagram of study selection.

### 3.2 Characteristics of included studies

Fourteen RCTs published between 2016 and 2025 were included. The studies were conducted in India, Iran, Thailand, Egypt, South Korea, and Türkiye and comprised nine two-arm RCTs, four three-arm RCTs, and one four-arm RCT. Analysed sample sizes ranged from 18 to 56 participants. Most studies enrolled adolescents or young adults; study-specific age information is reported in **Table 1**. Seven studies reported sex distribution: six included both female and male participants, whereas one enrolled women only. Six studies described participants as asymptomatic or free of relevant pain; symptom status was not consistently reported in the remaining studies.

**Table 1:** Characteristics of included studies.

Study	Participants	Intervention and comparator	Dose	Outcomes
(Moghadasi, 2025)	Randomised: 44 (11 per arm). Analysed: 41 (CCE 11; IFM+EFM 11; IFM 10; control 9). Age: 18–20 years (range). Sex and symptom status NR. Eligibility: FPI 6–12; rigid flatfoot excluded (DB).	CM. I: CCE (intrinsic and extrinsic foot-muscle, hip and core exercises). C: intrinsic + extrinsic foot-muscle exercises. Other arms: intrinsic exercise; no exercise.	12 wk; 3 supervised + 3 home sessions/wk; reported sessions progressed from 30–45 to 60–75 min.	FPI-6
(Ali et al., 2025)	Randomised: 44 (22/22). Analysed: 44 (22/22). 10 M/34 F. Age: 20.27 ± 0.98/19.91 ± 0.81 y. Symptom status NR. Eligibility: NDT >10 mm (DB).	PM. I: Pilates + SFE. C: SFE.	5 wk; Pilates 2 supervised sessions/wk, 30 min/session; daily home SFE in both groups.	Platform dynamic balance; FFI.
(Esen et al., 2025)	Randomised: 61 (HEG 20; FEG 21; CG 20). Analysed: 56 (18/19/19); 18 M/38 F. Median age (IQR): HEG 20.5 (20–22); FEG 20 (19–21); CG 21 (19–22) y. No recent pain/fatigue. Eligibility: NDT ≥10 mm; FPI-6 ≥6; positive Jack test (DA).	PM. I (HEG): clam exercise + SFE. C (CG): SFE. Other arm (FEG): three-dimensional foot–ankle exercise + SFE.	6 wk; added exercises 3 supervised sessions/wk; daily SFE.	NDT; FPI-6; footprint angles and indices.
(Raghav et al., 2024)	Randomised: 54 (18/18/18). Analysed: 54 (18/18/18). Sex NR. Eligible age: 18–30 y; group ages NR. Asymptomatic athletes. Eligibility: NDT >10 mm; positive FPI (DB).	PM. I1: gluteus maximus strengthening + SFE. I2: gluteus medius strengthening + SFE. C: SFE.	4 wk; 6 home sessions/wk; remote checks 3 sessions/wk.	NDT; FPI-6; Stork test; modified SEBT.
(Zarali and Raeisi, 2023; Zarali et al., 2024)	Randomised: 45 (15 per arm). Analysed: 2024, 45 (15 per arm); 2023, 40 (I 13; C 13; CE 14). All F. Age in 2024: I 21.40 ± 1.12; C 22.26 ± 1.66; CE 21.93 ± 1.38 y. Symptom status NR. Eligibility: NDT >10 mm (DB).	CM. I: SFE + isometric hip abduction. C: SFE. Other arm (CE): combined strengthening, stretching and balance exercises.	6 wk; 3 supervised + 3 home sessions/wk.	NDT; stance/pressure and COP sway; YBT; joint-position sense.
(Brijwasi and Borkar, 2023)	Randomised: 52 (26/26). Analysed at 6 wk: 49 (25/24). Sex and symptom status NR. Baseline age: 21.1 ± 0.4/20.5 ± 0.9 y (randomised sample). Eligibility: NDT >10 mm; arch angle <130° (DB).	UM. I: ankle motion + SFE + gluteal strengthening + calf stretching. C: ankle motion + calf stretching.	6 wk; 3 sessions/wk; I 30 min/session; C approximately 8 min/session.	Navicular drop height; medial longitudinal arch angle.
(Engkananuwat and Kanlayanaphotporn, 2023)	Randomised: 52 (26/26). Analysed: 52 (26/26); 104 feet. 26 M/26 F. Age: 28.73 ± 4.61/30.03 ± 4.36 y. Asymptomatic. Eligibility: NDT >10 mm (DB).	CM. I: foot adduction, supination and SFE + gluteus medius strengthening. C: the same foot exercises.	8 wk; 5 home sessions/wk; weekly remote contact.	NDT; arch height index; muscle strength; plantar pressure; static displacement; multidirectional reach.
(Utsahachant et al., 2023)	Randomised and analysed: 45 (15 per arm); 15 M/30 F. Median age (IQR): I 21 (20–22); C 21 (21–29); no exercise 21 (21–26) y. No recent pain/fatigue. Eligibility: NDT >10 mm (DB).	PM. I: SFE integrated with wall squat and unilateral wall squat. C: SFE. Other arm: no exercise.	6 wk; daily home exercise; video contact 3 sessions/wk.	NDT; intrinsic foot-muscle test; foot kinematics; CPEI during gait.
(Saleh et al., 2022)	Randomised: 44 (22/22). Analysed: 44 (22/22); 7 M/37 F. Age: 21.09 ± 1.87/21.86 ± 3.80 y. Symptom status NR. Eligibility: FPI + 6 to +9; positive tiptoe test (DA).	PM. I: backward walking + SFE, towel curls, heel raises and calf stretching. C: the same local exercises. Walking sessions also included warm-up/cool-down movements and stretches.	6 wk; 3 sessions/wk; backward walking 30 min/session. Total comparator session time NR.	FPI-6; Limits of Stability.
(Sawant and Shinde, 2021)	Randomised: 40 (20/20), as reported. Analysed: 40 (20/20). Sex and symptom status NR. Overall mean age 21 y; reported range 18–25 y. Eligibility: NDT >10 mm; PAI >1.15 (DB).	PM. I: proximal-to-distal imbalance correction + intrinsic foot-muscle exercises. C: intrinsic foot-muscle exercises.	6 wk; exercises progressed after 3 wk. Weekly frequency and session duration NR.	NDT; Plantar Arch Index.
(Alam et al., 2019)	Randomised: 28 (14/14). Analysed: 28 (14/14). Sex NR. Age: 22.6 ± 2.37/21.4 ± 1.78 y. Asymptomatic. Eligibility: NDT >10 mm; iliopsoas tightness (DB).	UM. I: tibialis posterior strengthening + iliopsoas stretching and strengthening + towel curls. C: towel curls. Both groups received moist heat; shoes with insoles were used during tibialis posterior training.	6 wk; selective exercises 3 sessions/wk; daily towel curls in both groups.	NDT; YBT; tibialis anterior and abductor hallucis EMG.
(Mulchandani et al., 2017)	Randomised: 52 (26/26). Analysed: 52 (26/26). 7 M/45 F. Age: 20.03 ± 2.37/19.96 ± 1.86 y. Symptom status NR. Eligibility: NDT >10 mm; PAI >1.15 (DB).	CM. I: gluteal strengthening + intrinsic foot-muscle exercises. C: intrinsic foot-muscle exercises.	4 wk; 6 sessions/wk; session duration NR.	NDT; Plantar Arch Index.
(Goo et al., 2016b)	Randomised n NR. Reported group/analysis n: 18 (9/9). Sex and symptom status NR. Age: 21.89 ± 1.69/22.30 ± 2.06 y. Eligibility: NDT >10 mm (DB).	CM. I: gluteus maximus strengthening + toe-spread exercises. C: toe-spread exercises.	4 wk; 3 laboratory + 2 home sessions/wk.	NDT; lower-limb EMG during gait.
(Goo et al., 2016a)	Randomised n NR. Reported group/analysis n: 20 (10/10). Sex NR. Age: 21.8 ± 1.62/22.3 ± 1.95 y. No present or previous pain. Eligibility: NDT >10 mm (DB).	CM. I: hip external-rotator strengthening + toe-spread exercises. C: toe-spread exercises.	4 wk; 3 laboratory + 2 home sessions/wk.	NDT; lower-limb EMG during stair walking.

Paired group values are intervention/comparator. Age is mean ± SD unless labelled median (IQR), eligible age, or range. NR indicates that the specified information was not reported separately. CCE, comprehensive corrective exercise; CE, combined exercise; CPEI, centre-of-pressure excursion index; EMG, electromyography; F, female; FFI, foot function index; FPI-6, six-item foot posture index; IFM/EFM, intrinsic/extrinsic foot-muscle exercise; IQR, interquartile range; M, male; NDT, navicular drop test; RCT, randomised controlled trial; SEBT, star excursion balance test; SFE, short-foot exercise; YBT, Y-balance test; wk, week; y, year. I, intervention; C/CG, comparator/control group; HEG, hip-exercise group; FEG, foot-exercise group; COP, centre of pressure. Diagnostic reporting: DA, explicit flexibility test; DB, posture/navicular-drop threshold without a separately reported flexibility test. PAI, plantar arch index. CM, substantially matched background local programme; PM, partially matched programme or incompletely reported local-dose matching; UM, additional distinct local treatment components.

Diagnostic thresholds mainly involved navicular drop, FPI-6, or footprint indices. Two trials explicitly reported flexibility testing: the Jack test in Esen (Esen et al., 2025) and tiptoe testing in Saleh (Saleh et al., 2022). The remaining 12 trials did not separately describe a flexibility-confirmation test. The supplementary programme classification identified six CM, six PM, and two UM comparisons. The UM comparisons in Brijwasi and Alam (Alam et al., 2019; Brijwasi and Borkar, 2023) added local treatment components alongside proximal exercise. Proximal components included hip or gluteal strengthening, iliopsoas training, core/Pilates exercise, and closed-chain or gait-related tasks; local components included SFE, intrinsic-foot-muscle, toe-control, and foot–ankle exercises. Only eligible active-comparator contrasts contributed to the primary synthesis.

Intervention duration ranged from 4 to 12 weeks. Scheduled supervised or combined sessions ranged from two to six per week, while several studies prescribed additional daily home-based foot exercise. Delivery formats included supervised, home-based, and combined programmes, with remote or video follow-up used in several trials. Reported outcomes covered navicular drop; the Foot Posture Index; arch height, arch angle, or footprint-derived measures; balance and postural control; proprioception; muscle strength, muscle control, or electromyography; plantar pressure, gait, or lower-limb kinematics; and patient-reported foot-related symptoms or function. The characteristics of the included studies are summarised in **Table 1**.

### 3.3 Risk of bias

The RoB 2 assessments are presented in **Figure 3**. Overall, 3/14 studies (21.4%) were judged to be at low risk of bias, 8/14 (57.1%) were judged as having some concerns, and 3/14 (21.4%) were judged to be at high risk. No individual domain received a high-risk judgement. The two linked Zarali reports had identical judgements and were represented once in the study-level summary.

**Figure 3:**
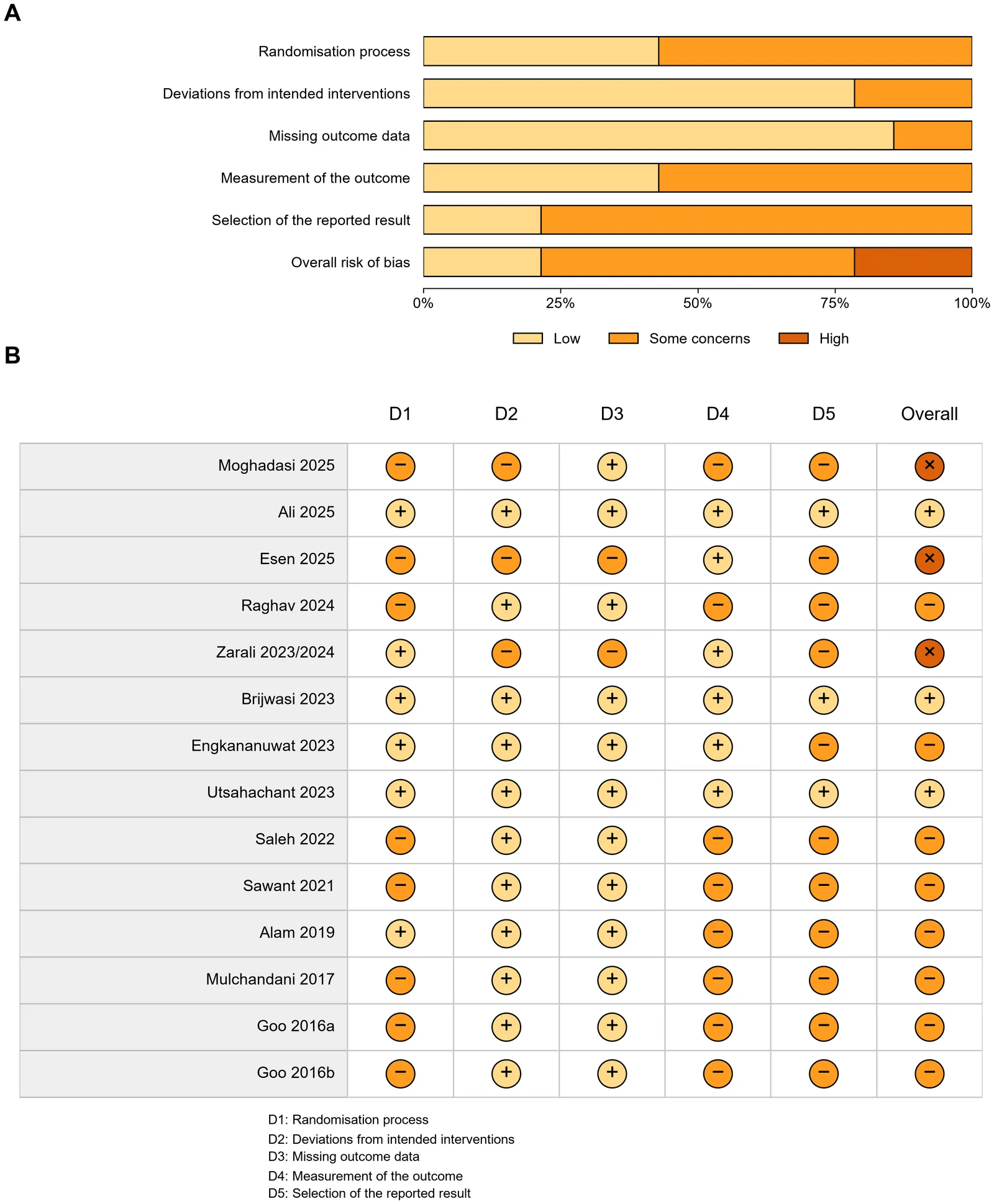
Risk-of-bias assessment using RoB 2: **(A)** distribution of domain-level judgements and **(B)** study-level judgements.

Bias in selection of the reported result was the most frequent source of concern, with 11/14 studies raising some concerns in this domain. Concerns were also common for the randomisation process and outcome measurement (8/14 studies for each), whereas fewer studies raised concerns regarding deviations from intended interventions (3/14) or missing outcome data (2/14).

### 3.4 Effects on static foot-posture outcomes

#### 3.4.1 Navicular drop

Eleven trials involving 410 participants contributed to the navicular-drop meta-analysis. The pooled estimate favoured the intervention (MD −2.34 mm, 95% CI −3.30 to −1.37; I²=84.6%; **Figure 4**).

**Figure 4:**
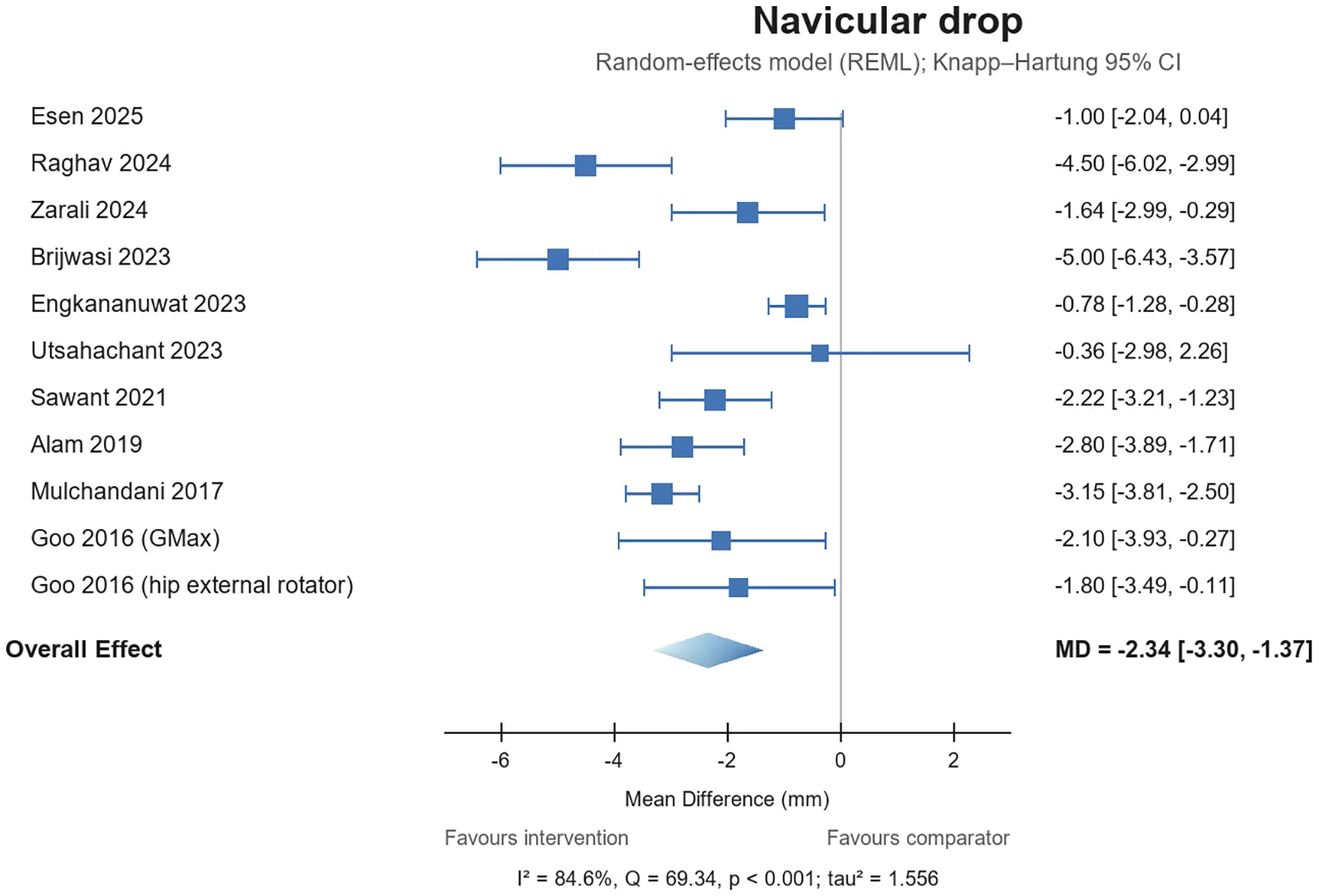
Effect of exercise programmes incorporating proximal kinetic-chain components on navicular drop. Negative mean differences favour the intervention.

#### 3.4.2 Foot posture index

Four trials involving 157 participants contributed to the Foot Posture Index-6 (FPI-6) meta-analysis. The pooled estimate was −1.67 points (Knapp–Hartung 95% CI −3.36 to 0.01; p=0.051; I²=89.1%; **Figure 5**). The confidence interval included the null.

**Figure 5:**
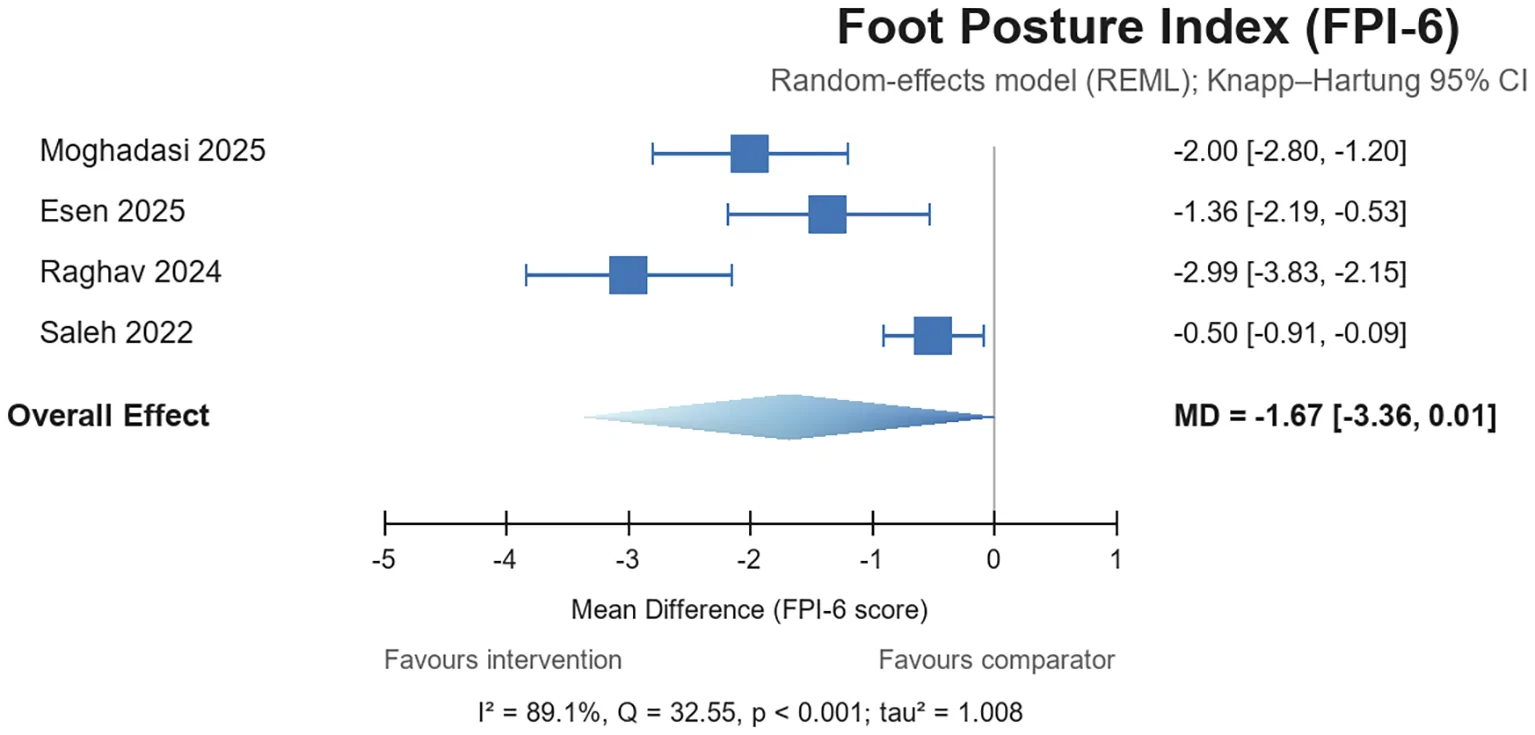
Effect of exercise programmes incorporating proximal kinetic-chain components on Foot Posture Index scores. Negative mean differences favour the intervention.

#### 3.4.3 Plantar arch index and other arch measures

Two trials involving 92 participants contributed to an exploratory meta-analysis of the Plantar Arch Index. The pooled estimate did not clearly favour either group (MD −0.134, 95% CI −0.278 to 0.009; I²=96.5%; **Figure 6**).

**Figure 6:**
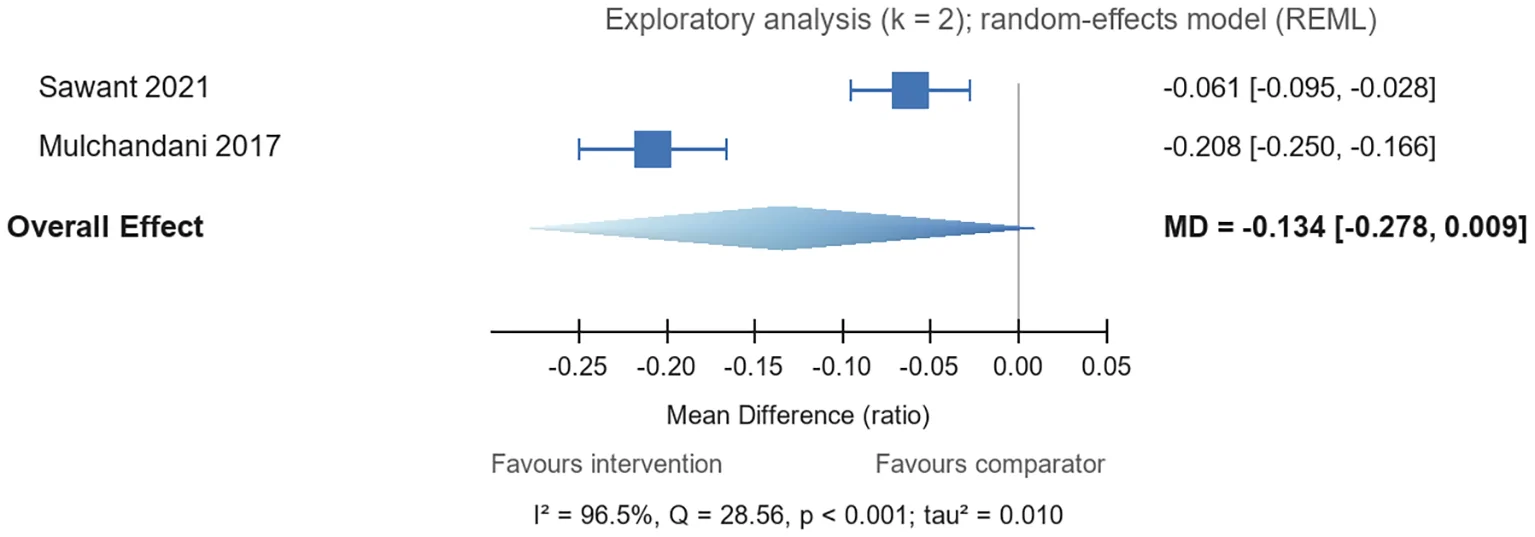
Exploratory meta-analysis of Plantar Arch Index outcomes. Negative mean differences favour the intervention.

At follow-up, the between-group difference in medial longitudinal arch angle was 20.00° (95% CI 15.47 to 24.53) (Brijwasi and Borkar, 2023), and the between-group difference in Arch Height Index was 0.010 (95% CI 0.006 to 0.014) (Engkananuwat and Kanlayanaphotporn, 2023). Post-intervention medians in the intervention and comparator groups were 30.49 and 30.35 for Clarke angle, 0.59 and 0.60 for Staheli Index, and 2.79 and 2.82 for Wejflog Index, respectively (Esen et al., 2025). Neither foot rotation (MD 0.45°, 95% CI −0.10 to 1.00) nor foot-surface area (MD −2.00 cm², 95% CI −10.35 to 6.35) showed a clear between-group difference (Zarali et al., 2024).

### 3.5 Balance and postural control

Two trials involving 54 participants contributed to exploratory Y-Balance Test meta-analyses. The pooled estimates were Hedges’ g=0.43 for anterior reach (95% CI −0.49 to 1.36; I²=65.1%), g=0.78 for posteromedial reach (95% CI −0.78 to 2.35; I²=86.5%), and g=0.66 for posterolateral reach (95% CI −1.18 to 2.50; I²=90.1%); all confidence intervals included the null (**Figure 7**). Pooled effects across the six quantitative outcomes are summarised in **Table 2**.

**Figure 7:**
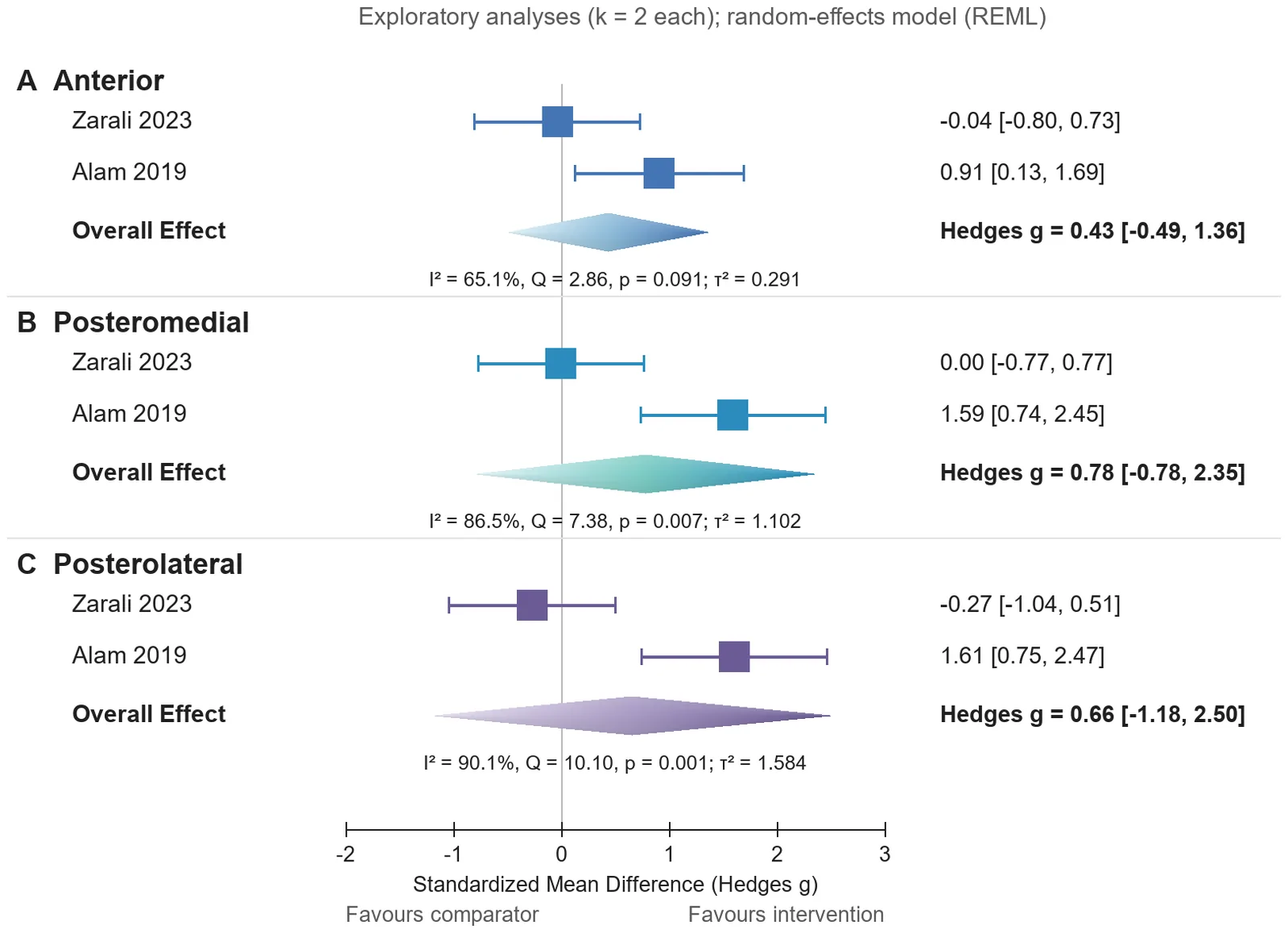
Exploratory meta-analyses of Y-Balance Test reach in the **(A)** anterior, **(B)** posteromedial, and **(C)** posterolateral directions. Positive Hedges’ g values favour the intervention.

**Table 2:** Pooled effects at the end of intervention.

Outcome	k/N	Measure	Value	95% CI	p	I² (%)
Navicular drop	11/410	MD (mm)	−2.34	−3.30 to −1.37	<0.001	84.6
FPI-6	4/157	MD (points)	−1.67	−3.36 to 0.01	0.051	89.1
Plantar Arch Index	2/92	MD (ratio)	−0.134	−0.278 to 0.009	0.067	96.5
Y-Balance anterior	2/54	Hedges g	0.43	−0.49 to 1.36	0.358	65.1
Y-Balance posteromedial	2/54	Hedges g	0.78	−0.78 to 2.35	0.326	86.5
Y-Balance posterolateral	2/54	Hedges g	0.66	−1.18 to 2.50	0.480	90.1

k/N denotes independent trials/analysed participants for each outcome; participants are not summed across outcomes. Estimates compare intervention-end values (intervention minus comparator). Negative MDs favour intervention for posture measures; positive Hedges g values favour intervention for Y-Balance reach.

Among the other balance outcomes, between-group differences in single-leg Stork time ranged from 2.66 to 6.16 s across the intervention comparisons (Raghav et al., 2024), while anteroposterior and mediolateral displacement were lower by 0.49 and 0.45 cm, respectively (Engkananuwat and Kanlayanaphotporn, 2023). Directional platform-deviation scores were 0.71–1.01 points lower (Ali et al., 2025), and modified Star Excursion Balance Test composite-score values were 14.03–29.41 points higher (Raghav et al., 2024). The follow-up between-group difference in Limits of Stability was 7.40 percentage points (95% CI 3.75 to 11.05) (Saleh et al., 2022), whereas between-group differences in multidirectional reach included the null (Engkananuwat and Kanlayanaphotporn, 2023). Of five centre-of-pressure sway measures, only mediolateral sway excluded the null (MD −17.87 mm, 95% CI −34.80 to −0.94); the remaining estimates included the null (Zarali et al., 2024).

### 3.6 Other clinical, biomechanical, and patient-reported outcomes

Proprioceptive and muscle-related outcomes were reported using joint-position-sense, strength, intrinsic-foot-muscle, and electromyographic measures. Joint-position-sense error did not clearly differ between groups (MD 0.23°, 95% CI −1.12 to 1.58), and the estimates for ankle dorsiflexion and plantar-flexion strength also included the null (Zarali and Raeisi, 2023). One trial reported greater gluteus medius strength with the proximal-plus-foot programme (MD 12.81 N, 95% CI 5.76 to 19.86), whereas tibialis posterior and flexor hallucis brevis strength estimates included the null (Engkananuwat and Kanlayanaphotporn, 2023). An ordinal intrinsic-foot-muscle test showed little between-group separation (Utsahachant et al., 2023), and electromyographic findings varied across muscles and gait or stair phases (Goo et al., 2016b; Goo et al., 2016a; Alam et al., 2019).

Plantar-pressure, gait, and kinematic outcomes were reported across several trials. Medial forefoot pressure was lower in the intervention group (MD −8.61 kPa, 95% CI −13.42 to −3.80), whereas estimates for the medial hindfoot and other plantar regions included the null (Engkananuwat and Kanlayanaphotporn, 2023). The between-group estimate for maximum pressure was also imprecise (MD −0.10 N, 95% CI −1.47 to 1.27) (Zarali et al., 2024). Gait-derived medial-longitudinal-arch timing and angles, midfoot eversion excursion, and centre-of-pressure excursion showed small or imprecise between-group contrasts (Utsahachant et al., 2023). Estimates for hallux-valgus and fifth-toe-varus angles included the null (Esen et al., 2025).

Patient-reported foot function was assessed in one trial, in which the Foot Function Index favoured the proximal-plus-foot intervention at 5 weeks (MD −8.55 percentage points, 95% CI −11.47 to −5.63) (Ali et al., 2025). Reported adherence was 85.7% and 88.6% in that trial (Ali et al., 2025), 95% in both relevant groups in another trial (Esen et al., 2025), and 35.7 and 36.2 of 40 prescribed days in a third (Engkananuwat and Kanlayanaphotporn, 2023). One trial reported no adverse events among participants who completed follow-up (Brijwasi and Borkar, 2023). Another reported three withdrawals because of discomfort, although group-specific safety denominators were unavailable (Esen et al., 2025). Individual-trial estimates and descriptive outcomes are tabulated separately in **Supplementary Material S3**.

### 3.7 Sensitivity analysis

Change-score analyses at r=0.30 and r=0.70 yielded navicular-drop MDs of −2.28 mm (95% CI −3.05 to −1.51) and −2.25 mm (95% CI −3.01 to −1.48), respectively. Corresponding FPI-6 estimates were −1.76 points (95% CI −3.50 to −0.02; p=0.0486) and −1.79 points (95% CI −3.50 to −0.09; p=0.0444). Unlike the primary FPI-6 interval, these intervals narrowly excluded the null. Excluding Moghadasi (2025) yielded an FPI-6 MD of −1.58 points (3 trials; 95% CI −4.73 to 1.56). Navicular-drop estimates remained below zero after excluding Engkananuwat (Engkananuwat and Kanlayanaphotporn, 2023) (10 trials; MD −2.54 mm, 95% CI −3.53 to −1.55). Exploratory Plantar Arch Index intervals included the null at both correlations; the exploratory anterior Y-Balance interval excluded the null only at r=0.70. Full results are provided in **Supplementary Material S2**.

In the exploratory programme-matching analyses, the navicular-drop estimate was −1.99 mm after excluding UM comparisons (9 trials, 333 participants; 95% CI −2.95 to −1.04; I²=80.2%; τ²=1.106), compared with −2.34 mm in the primary analysis. Restriction to CM comparisons yielded an MD of −1.88 mm (5 trials, 172 participants; 95% CI −3.09 to −0.68; I²=79.0%; τ²=0.856). Separately excluding CM or PM comparisons yielded MDs of −2.72 mm (6 trials, 238 participants; 95% CI −4.59 to −0.85; I²=85.3%; τ²=2.469) and −2.44 mm (7 trials, 249 participants; 95% CI −3.71 to −1.17; I²=86.0%; τ²=1.538), respectively. Thus, the direction persisted across these restrictions, but substantial heterogeneity remained.

For FPI-6, the CM-only restriction retained only Moghadasi (22 participants; single-trial MD −2.00 points, 95% CI −2.80 to −1.20); excluding CM retained three PM trials (135 participants; MD −1.58 points, 95% CI −4.73 to 1.56). Neither FPI-6 nor Plantar Arch Index included a UM comparison, so excluding UM reproduced their primary estimates without providing a new check. CM-only Plantar Arch Index retained Mulchandani (52 participants; single-trial MD −0.208, 95% CI −0.250 to −0.166). For each exploratory Y-Balance direction, retaining CM or excluding UM left only Zarali (26 participants), with single-trial intervals including the null; excluding CM left only Alam (28 participants). Scenario membership, effect estimates and heterogeneity statistics are provided in **Supplementary Material S2**.

### 3.8 Certainty of evidence

The certainty of evidence ranged from very low to low across the quantitative outcomes (**Table 3**). Navicular-drop evidence was rated as low because of risk of bias and unexplained variation in effect magnitude. FPI-6 and exploratory Plantar Arch Index evidence was rated as very low because of risk of bias, inconsistency, and imprecision. Evidence for all three exploratory Y-Balance Test directions was also very low. Outcome-specific reasons for downgrading and for retaining domains without downgrading are provided in **Supplementary Material S4**.

**Table 3:** GRADE evidence profile for the principal quantitative outcomes.

Outcome	Risk of bias	Inconsistency	Indirectness	Imprecision	Publication bias	Certainty
Navicular drop (11 RCTs; n=410)	Serious (−1)	Serious (−1)	Not serious	Not serious	Not downgraded	Low
Foot Posture Index (4 RCTs; n=157)	Serious (−1)	Serious (−1)	Not serious	Serious (−1)	Not downgraded	Very low
Plantar Arch Index (2 RCTs; n=92)	Serious (−1)	Serious (−1)	Not serious	Serious (−1)	Not downgraded	Very low
Y-Balance—anterior (2 RCTs; n=54)	Serious (−1)	Serious (−1)	Not serious	Serious (−1)	Not downgraded	Very low
Y-Balance—posteromedial (2 RCTs; n=54)	Serious (−1)	Serious (−1)	Not serious	Very serious (−2)	Not downgraded	Very low
Y-Balance—posterolateral (2 RCTs; n=54)	Serious (−1)	Serious (−1)	Not serious	Very serious (−2)	Not downgraded	Very low

Randomised evidence started at high certainty; −1 and −2 denote downgrading by one and two levels. Judgements concern short-term measured outcomes and whole-programme comparisons within the eligible population, not isolated proximal effects or patient-important benefit. Inconsistency reflects unexplained differences in magnitude, not I² alone. For plantar arch index, concerns about allocation, measurement, and selective reporting involved both contributing trials.

## 4 Discussion

### 4.1 Principal findings

Exercise programmes incorporating proximal kinetic-chain components may reduce short-term navicular drop compared with local or basic foot–ankle exercise programmes. This programme-level finding is supported by low-certainty evidence, with substantial heterogeneity limiting confidence in its magnitude. FPI-6 effects remain uncertain: the primary confidence interval included zero, whereas change-score intervals only narrowly excluded it. The exploratory Plantar Arch Index and Y-Balance analyses were also inconclusive. Evidence for functional, neuromuscular, gait, and patient-reported outcomes was sparse and mainly derived from individual trials.

In exploratory programme-matching analyses added during revision, restricting navicular-drop synthesis to CM comparisons retained a negative estimate (MD −1.88 mm), although heterogeneity remained substantial (I²=79.0%) and Engkananuwat and Mulchandani contributed 52.0% of the weight. Together with the exclusion-of-UM analysis, this suggests that the overall direction was not solely dependent on comparisons adding distinct local treatments. Matching of local exercise content did not establish equivalent total exercise dose, supervision, or contact time, so these findings concern programme comparisons rather than an isolated proximal effect. Sparse categories and the absence of an interaction test also limit interpretation of differences between analysis scenarios.

### 4.2 Static foot-posture responses

The navicular-drop MD of −2.34 mm represents a between-group difference in a static foot-posture measure at the end of intervention, not a demonstrated improvement in pain, function, participation, or quality of life. Substantial heterogeneity (I²=84.6%) limits the generalisability of its magnitude. FPI-6 was also highly heterogeneous (I²=89.1%), with an uncertain primary estimate. Navicular drop measures landmark displacement between test conditions, whereas FPI-6 combines six observations of standing posture (Vinicombe et al., 2001; Redmond et al., 2006; Keenan et al., 2007). Footprint indices describe plantar-contact geometry (Cavanagh and Rodgers, 1987), and Arch Height Index relates dorsum height to truncated foot length (Williams and McClay, 2000). These different constructs need not respond uniformly to exercise.

Statistical significance, measurement error, and patient-important change address different questions. In healthy young adults, Fraser et al. reported an FPI standard error of measurement of 1.6 points and a minimal detectable change (MDC) of 4.5 points (Fraser et al., 2017). This protocol-specific MDC is an individual-change threshold, not a minimal clinically important difference (MCID), and cannot be applied directly to a pooled between-group mean difference. Navicular-drop reliability also depends on the measurement procedure (Vinicombe et al., 2001). No directly applicable, validated patient-anchored threshold was identified for these pooled effects; their clinical importance therefore remains uncertain. This distinction is particularly relevant when a low arch is an asymptomatic morphological variant (Mosca, 2010; Halabchi et al., 2013). Associations between pronated posture and selected overuse outcomes (Neal et al., 2014) provide context but are not evidence that exercise-related postural change prevents injury.

### 4.3 Neuromuscular and mechanical responses

The isolated gluteus medius strength improvement was compatible with training specificity, while other strength and proprioceptive estimates were imprecise or showed little between-group separation. Muscle- and task-specific electromyographic responses describe activation rather than force production or global motor control; neither increased nor decreased amplitude is inherently beneficial. Acute intrinsic-foot-muscle T2 responses provide exercise-activation context, not evidence of longitudinal adaptation (Gooding et al., 2016). Lower medial forefoot pressure may similarly reflect regional load redistribution rather than overall unloading. These findings provide outcome-specific observations, but little direct evidence linking proximal adaptation to distal postural change.

Foot-core and induced-pronation studies support mechanical coupling within the lower limb (McKeon et al., 2015; Resende et al., 2015; Hornestam et al., 2021). Stroke studies additionally describe altered foot sensation, posture and function (Kunkel et al., 2017), and ankle stiffness and tissue compliance (Rydahl and Brouwer, 2004). These neurological observations offer indirect context for interactions among sensory input, tissue mechanics, and ankle mobility. They neither identify the mechanism of the reviewed exercise programmes nor justify extending their treatment estimates to neurological populations.

### 4.4 Dynamic postural control, gait, and patient-reported outcomes

Each exploratory Y-Balance direction was supported by only two trials, with primary confidence intervals including zero and very-low-certainty evidence. These results are inconclusive, rather than evidence of no effect. Other balance tests assessed different tasks, and gait timing, arch angles, and centre-of-pressure measures were sparse or imprecise. Static foot-posture change should therefore remain distinct from demonstrated functional improvement or injury prevention.

Orthotic research provides complementary sensory-mechanical context: customised 3D-printed insoles have been evaluated using balance and performance outcomes (Öztürk and Kurul, 2024), while customised and prefabricated insoles have been compared for plantar-pressure and comfort responses (Xu et al., 2019). These external foot interventions illustrate alternative pathways to dynamic responses, not evidence of a proximal-exercise mechanism. Patient-reported foot function was assessed in only one short trial, leaving the relationship between measured postural responses and patient-perceived benefit insufficiently characterised.

### 4.5 Strengths, limitations, and research priorities

Active exercise comparators and trial-level linkage of related reports strengthened the review’s programme-level interpretation. Diagnostic verification nevertheless remained limited: only two trials explicitly reported flexibility testing. The other 12 trials did not separately report a flexibility-confirmation test, so we could not verify that all participants met a common definition of flexible flatfoot. This diagnostic reporting gap raises the possibility of population misclassification and limits the applicability of the findings to confirmed flexible flatfoot.

Multicomponent interventions and incomplete matching or reporting of dose, progression, supervision, and contact time constrained component attribution. Even CM comparisons did not establish equal total exposure. The *post hoc* category analyses contained few trials, and CM-only FPI-6, Plantar Arch Index, and Y-Balance checks each retained only one trial. Substantial heterogeneity persisted in CM-only navicular drop, alongside small samples and risk-of-bias concerns across the evidence base. Statistical conversions and assumptions about bilateral dependence and pre–post correlations added uncertainty; FPI-6 interval interpretation and exploratory anterior Y-Balance findings varied with analysis choices. Four unretrieved reports introduced potential availability bias of unknown direction.

Participants were predominantly young and partly asymptomatic, limiting applicability to symptomatic and older clinical populations. Interventions lasted 4–12 weeks, with little evidence on maintenance after training ended. Sparse functional and patient-reported outcomes and incomplete adherence and adverse-event reporting further restricted assessment of clinical usefulness and harms.

Future trials should explicitly verify flexibility and use the same background local programme, with matched or transparently documented total dose, progression, supervision, and contact time. Adequately powered trials in symptomatic populations, including older adults, should extend follow-up and prespecify patient-important and dynamic functional outcomes. Concurrent repeated measures of proximal capacity, lower-limb kinematics, foot posture, and function could test whether postural responses mediate meaningful benefit. Exercise delivery, adherence, and harms should be reported consistently using CERT and CONSORT Harms guidance (Slade et al., 2016; Junqueira et al., 2023).

## 5 Conclusion

In the studied low-arch/pronated-foot populations, programmes incorporating proximal kinetic-chain components may reduce short-term navicular drop compared with local or basic foot–ankle exercise, although substantial heterogeneity limits confidence in the effect magnitude. FPI-6, Plantar Arch Index, and dynamic outcomes remain uncertain. Trials with matched local training and total exposure, symptomatic participants, longer follow-up, and prespecified functional and patient-reported outcomes are needed to clarify whether short-term postural responses translate into meaningful clinical benefit.

## Data Availability

The original contributions presented in the study are included in the article/**Supplementary Material**. Further inquiries can be directed to the corresponding author.
