# Supplementary material for: Exercise programmes incorporating proximal kinetic-chain components in flexible flatfoot: a systematic review and meta-analysis of foot posture, neuromuscular, and dynamic outcomes
Source: Front Physiol. 2026 Oct 12;17:1959545. doi: 10.3389/fphys.2026.1959545 (PMC13696242; doi:10.3389/fphys.2026.1959545)
Supplement: Supplementary file 1 [file Table1.docx]

**Supplementary Material 1. Full Search Strategies**

**Review title: Exercise Programmes Incorporating Proximal Kinetic-Chain Components in Flexible Flatfoot: A Systematic Review and Meta-analysis of Foot Posture, Neuromuscular, and Dynamic Outcomes**

This supplement reports the complete strategies used to identify studies for the review. The four primary electronic databases were searched from inception through the dates shown below. No language, publication-date, publication-status, or document-type limits were applied. The four primary searches yielded 6,543 records before deduplication. Supplementary searches were conducted separately and were not added to the 6,543-record primary-database total.

**Table S1. Primary electronic database searches**

| **Database and platform** | **Search date** | **Coverage** | **Limits** | **Records** |
| --- | --- | --- | --- | --- |
| PubMed (NCBI PubMed) | 19 Jul 2026 | Inception to search date | None | 1,339 |
| Web of Science Core Collection (Clarivate) | 19 Jul 2026 | Inception to search date | None | 1,622 |
| Scopus (Elsevier) | 19 Jul 2026 | Inception to search date | None | 2,760 |
| SPORTDiscus with Full Text (EBSCOhost) | 19 Jul 2026 | Inception to search date | None | 822 |
| **Total** |  |  |  | **6,543** |

**S1. PubMed**

**Database and platform:** PubMed, National Center for Biotechnology Information.

**Search date:** 19 July 2026 (Asia/Shanghai, UTC+08:00).

**Coverage:** Database inception to the search date.

**Limits and filters:** None.

**Records retrieved:** 1,339.

**Exact search strategy**

(

"Flatfoot"[Mesh]

OR flatfoot*[tiab]

OR "flat foot"[tiab:~0]

OR "flat feet"[tiab:~0]

OR "flexible flatfoot"[tiab:~0]

OR "flexible flat foot"[tiab:~0]

OR "flexible pes planus"[tiab:~0]

OR "pes planus"[tiab:~0]

OR "pes planovalgus"[tiab:~0]

OR planovalgus[tiab]

OR "low arch"[tiab:~0]

OR "low arches"[tiab:~0]

OR "low arched foot"[tiab:~0]

OR "low arched feet"[tiab:~0]

OR "fallen arch"[tiab:~0]

OR "fallen arches"[tiab:~0]

OR "collapsed arch"[tiab:~0]

OR "collapsed arches"[tiab:~0]

OR "pronated foot"[tiab:~0]

OR "pronated feet"[tiab:~0]

OR overpronat*[tiab]

OR "excessive pronation"[tiab:~0]

OR ("medial longitudinal arch"[tiab:~0] AND (low*[tiab] OR reduc*[tiab] OR flatten*[tiab] OR collapse*[tiab]))

)

AND

(

"Exercise Therapy"[Mesh]

OR "Resistance Training"[Mesh]

OR exercise*[tiab]

OR train*[tiab]

OR strengthen*[tiab]

OR rehabilitat*[tiab]

OR "corrective exercise"[tiab:~0]

OR "therapeutic exercise"[tiab:~0]

OR "neuromuscular training"[tiab:~0]

OR "motor control"[tiab:~0]

OR "home exercise"[tiab:~0]

OR "short foot"[tiab:~0]

OR short-foot[tiab]

OR "foot core"[tiab:~0]

OR foot-core[tiab]

OR "intrinsic foot muscle"[tiab:~0]

OR "intrinsic foot muscles"[tiab:~0]

OR "intrinsic muscle training"[tiab:~0]

OR "doming exercise"[tiab:~0]

OR "arch lift"[tiab:~0]

OR "toe yoga"[tiab:~0]

OR "toe curl"[tiab:~0]

OR "toe curls"[tiab:~0]

OR "toe flexion"[tiab:~0]

OR "toe spreading"[tiab:~0]

OR "toe abduction"[tiab:~0]

OR "toe exercise"[tiab:~0]

OR "foot exercise"[tiab:~0]

OR "foot exercises"[tiab:~0]

OR "foot strengthening"[tiab:~0]

OR "ankle exercise"[tiab:~0]

OR "ankle exercises"[tiab:~0]

OR "foot ankle exercise"[tiab:~0]

OR "foot ankle exercises"[tiab:~0]

OR "three dimensional foot ankle exercise"[tiab:~0]

OR "tibialis posterior"[tiab:~0]

OR "posterior tibial"[tiab:~0]

OR "peroneus longus"[tiab:~0]

OR "fibularis longus"[tiab:~0]

OR "gluteus medius"[tiab:~0]

OR "gluteal strengthening"[tiab:~0]

OR "hip abductor"[tiab:~0]

OR "hip abductors"[tiab:~0]

OR "hip external rotator"[tiab:~0]

OR "hip external rotators"[tiab:~0]

OR "clam exercise"[tiab:~0]

OR "clamshell exercise"[tiab:~0]

OR "hip focused exercise"[tiab:~0]

OR "hip strengthening"[tiab:~0]

OR "lower extremity exercise"[tiab:~0]

OR "lower extremity exercises"[tiab:~0]

OR "lower limb exercise"[tiab:~0]

OR "lower limb exercises"[tiab:~0]

OR "closed kinetic chain"[tiab:~0]

OR "kinetic chain"[tiab:~0]

OR multisegment*[tiab]

OR multi-segment*[tiab]

OR multijoint[tiab]

OR multi-joint[tiab]

OR "core stability"[tiab:~0]

OR "core stabilization"[tiab:~0]

OR "trunk exercise"[tiab:~0]

OR "comprehensive exercise"[tiab:~0]

OR "combined exercise"[tiab:~0]

OR "integrated exercise"[tiab:~0]

OR "whole lower limb"[tiab:~0]

OR Pilates[tiab]

)

**Execution note:** PubMed reported that the wildcard in low* was ignored because fewer than four characters preceded the asterisk. The submitted query was retained unchanged.

**S2. Web of Science Core Collection**

**Database and platform:** Web of Science Core Collection, Clarivate Web of Science.

**Search date:** 19 July 2026 (Asia/Shanghai, UTC+08:00).

**Coverage:** All years/database inception to the search date.

**Editions:** CCR-EXPANDED; IC; CPCI-S; SCI-EXPANDED; SSCI.

**Search field:** Topic (TS).

**Limits and filters:** None.

**Records retrieved:** 1,622.

**Exact search strategy**

TS=(

flatfoot*

OR "flat foot"

OR "flat feet"

OR "flexible flatfoot"

OR "flexible flat foot"

OR "flexible flat feet"

OR "flexible pes planus"

OR "pes planus"

OR "pes planovalgus"

OR planovalgus

OR (low NEAR/1 arch*)

OR (fallen NEAR/1 arch*)

OR (collapsed NEAR/1 arch*)

OR (pronat* NEAR/2 foot)

OR overpronat*

OR "excessive pronation"

OR

(

"medial longitudinal arch"

NEAR/5

(low* OR reduc* OR flatten* OR collapse*)

)

)

AND

TS=(

exercise*

OR train*

OR strengthen*

OR rehabilitat*

OR "corrective exercise"

OR "therapeutic exercise"

OR "neuromuscular training"

OR "motor control"

OR "home exercise"

OR "short foot"

OR "short-foot"

OR "foot core"

OR "foot-core"

OR ("intrinsic foot" NEAR/2 muscle*)

OR "intrinsic muscle training"

OR "doming exercise"

OR "arch lift"

OR "toe yoga"

OR

(

toe

NEAR/2

(curl* OR flex* OR spread* OR abduct* OR exercise* OR train*)

)

OR

(

foot

NEAR/2

(exercise* OR train* OR strengthen*)

)

OR

(

ankle

NEAR/2

(exercise* OR train* OR strengthen*)

)

OR "foot ankle exercise"

OR "foot-ankle exercise"

OR "three dimensional foot ankle exercise"

OR "three-dimensional foot-ankle exercise"

OR "tibialis posterior"

OR "posterior tibial"

OR "peroneus longus"

OR "fibularis longus"

OR "gluteus medius"

OR (glute* NEAR/2 strengthen*)

OR

(

hip

NEAR/3

(

abductor*

OR rotator*

OR strengthen*

OR exercise*

OR train*

OR neuromuscular

)

)

OR "clam exercise"

OR "clamshell exercise"

OR "hip focused exercise"

OR "hip-focused exercise"

OR "lower extremity exercise"

OR "lower extremity exercises"

OR "lower limb exercise"

OR "lower limb exercises"

OR "whole lower limb"

OR "closed kinetic chain"

OR "kinetic chain"

OR multisegment*

OR "multi-segment"

OR "multi-segmental"

OR multijoint

OR "multi-joint"

OR "core stability"

OR "core stabilization"

OR "trunk exercise"

OR "pelvic stability"

OR "comprehensive exercise"

OR "combined exercise"

OR "integrated exercise"

OR "corrective training"

OR Pilates

)

**S3. Scopus**

**Database and platform:** Scopus, Elsevier.

**Search date:** 19 July 2026 (Asia/Shanghai, UTC+08:00).

**Coverage:** Database inception to the search date.

**Search fields:** Title, abstract, and keywords (TITLE-ABS-KEY).

**Limits and filters:** None.

**Records retrieved:** 2,760.

**Exact search strategy**

TITLE-ABS-KEY(

flatfoot*

OR "flat foot"

OR "flat feet"

OR "flexible flatfoot"

OR "flexible flat foot"

OR "flexible flat feet"

OR "flexible pes planus"

OR "pes planus"

OR "pes planovalgus"

OR planovalgus

OR (low W/1 arch*)

OR (fallen W/1 arch*)

OR (collapsed W/1 arch*)

OR (pronat* W/2 foot)

OR overpronat*

OR "excessive pronation"

OR

(

"medial longitudinal arch"

W/5

(

low*

OR reduc*

OR flatten*

OR collapse*

)

)

)

AND

TITLE-ABS-KEY(

exercise*

OR train*

OR strengthen*

OR rehabilitat*

OR "corrective exercise"

OR "therapeutic exercise"

OR "neuromuscular training"

OR "motor control"

OR "home exercise"

OR "short foot"

OR "short-foot"

OR "foot core"

OR "foot-core"

OR ("intrinsic foot" W/2 muscle*)

OR "intrinsic muscle training"

OR "doming exercise"

OR "arch lift"

OR "toe yoga"

OR

(

toe

W/2

(

curl*

OR flex*

OR spread*

OR abduct*

OR exercise*

OR train*

)

)

OR

(

foot

W/2

(

exercise*

OR train*

OR strengthen*

)

)

OR

(

ankle

W/2

(

exercise*

OR train*

OR strengthen*

)

)

OR "foot ankle exercise"

OR "foot-ankle exercise"

OR "three dimensional foot ankle exercise"

OR "three-dimensional foot-ankle exercise"

OR "tibialis posterior"

OR "posterior tibial"

OR "peroneus longus"

OR "fibularis longus"

OR "gluteus medius"

OR (glute* W/2 strengthen*)

OR

(

hip

W/3

(

abductor*

OR rotator*

OR strengthen*

OR exercise*

OR train*

OR neuromuscular

)

)

OR "clam exercise"

OR "clamshell exercise"

OR "hip focused exercise"

OR "hip-focused exercise"

OR "lower extremity exercise"

OR "lower extremity exercises"

OR "lower limb exercise"

OR "lower limb exercises"

OR "whole lower limb"

OR "closed kinetic chain"

OR "kinetic chain"

OR multisegment*

OR "multi-segment"

OR "multi-segmental"

OR multijoint

OR "multi-joint"

OR "core stability"

OR "core stabilization"

OR "trunk exercise"

OR "pelvic stability"

OR "comprehensive exercise"

OR "combined exercise"

OR "integrated exercise"

OR "corrective training"

OR Pilates

)

**S4. SPORTDiscus with Full Text**

**Database and platform:** SPORTDiscus with Full Text, EBSCOhost.

**Search date:** 19 July 2026 (Asia/Shanghai, UTC+08:00).

**Coverage:** Database inception to the search date.

**Search mode:** Boolean search.

**Limits and filters:** None observed.

**Records retrieved:** 822.

**Query terms entered**

(

flatfoot*

OR "flat foot"

OR "flat feet"

OR "flexible flatfoot"

OR "flexible flat foot"

OR "flexible flat feet"

OR "flexible pes planus"

OR "pes planus"

OR "pes planovalgus"

OR planovalgus

OR (low N1 arch*)

OR (fallen N1 arch*)

OR (collapsed N1 arch*)

OR (pronat* N2 foot)

OR overpronat*

OR "excessive pronation"

OR

(

"medial longitudinal arch"

N5

(

low*

OR reduc*

OR flatten*

OR collapse*

)

)

)

AND

(

exercise*

OR train*

OR strengthen*

OR rehabilitat*

OR "corrective exercise"

OR "therapeutic exercise"

OR "neuromuscular training"

OR "motor control"

OR "home exercise"

OR "short foot"

OR short-foot

OR "foot core"

OR foot-core

OR ("intrinsic foot" N2 muscle*)

OR "intrinsic muscle training"

OR "doming exercise"

OR "arch lift"

OR "toe yoga"

OR (toe N2 (curl* OR flex* OR spread* OR abduct* OR exercise* OR train*))

OR (foot N2 (exercise* OR train* OR strengthen*))

OR (ankle N2 (exercise* OR train* OR strengthen*))

OR "foot ankle exercise"

OR "foot-ankle exercise"

OR "three dimensional foot ankle exercise"

OR "three-dimensional foot-ankle exercise"

OR "tibialis posterior"

OR "posterior tibial"

OR "peroneus longus"

OR "fibularis longus"

OR "gluteus medius"

OR (glute* N2 strengthen*)

OR (hip N3 (abductor* OR rotator* OR strengthen* OR exercise* OR train* OR neuromuscular))

OR "clam exercise"

OR "clamshell exercise"

OR "hip focused exercise"

OR "hip-focused exercise"

OR "lower extremity exercise"

OR "lower extremity exercises"

OR "lower limb exercise"

OR "lower limb exercises"

OR "whole lower limb"

OR "closed kinetic chain"

OR "kinetic chain"

OR multisegment*

OR multi-segment*

OR multijoint

OR multi-joint

OR "core stability"

OR "core stabilization"

OR "trunk exercise"

OR "pelvic stability"

OR "comprehensive exercise"

OR "combined exercise"

OR "integrated exercise"

OR "corrective training"

OR Pilates

)

**S5. Europe PMC supplementary search**

**Database and platform:** Europe PMC, Europe PMC REST API.

**Search date:** 18 July 2026.

**Coverage:** Database inception to the search date.

**Search fields:** Title and abstract (TITLE_ABS).

**Limits and filters:** None.

**Records retrieved:** 1,137.

**Exact search strategy**

TITLE_ABS:(flatfoot* OR "flat foot" OR "flat feet" OR "flexible flatfoot" OR "flexible flat foot" OR "flexible pes planus" OR "pes planus" OR "pes planovalgus" OR planovalgus OR "low arch" OR "low arched foot" OR "fallen arch" OR "collapsed arch" OR "pronated foot" OR "pronated feet" OR overpronat* OR "excessive pronation" OR "medial longitudinal arch") AND TITLE_ABS:(exercise* OR train* OR strengthen* OR rehabilitat* OR "corrective exercise" OR "therapeutic exercise" OR "neuromuscular training" OR "motor control" OR "short foot" OR "foot core" OR "intrinsic foot muscle" OR "doming exercise" OR "arch lift" OR "toe yoga" OR "toe curl" OR "toe exercise" OR "foot exercise" OR "foot strengthening" OR "ankle exercise" OR "foot ankle exercise" OR "tibialis posterior" OR "posterior tibial" OR "peroneus longus" OR "fibularis longus" OR "gluteus medius" OR "gluteal strengthening" OR "hip abductor" OR "hip strengthening" OR "clam exercise" OR "clamshell exercise" OR "lower extremity exercise" OR "lower limb exercise" OR "closed kinetic chain" OR "kinetic chain" OR multisegment* OR multijoint OR "core stability" OR "trunk exercise" OR "comprehensive exercise" OR "combined exercise" OR "integrated exercise" OR Pilates)

**S6. ClinicalTrials.gov supplementary search**

**Registry and platform:** ClinicalTrials.gov, API v2.

**Search date:** 18 July 2026.

**Coverage:** Registry inception to the search date.

**Limits and filters:** None.

**Unique registry records retrieved:** 80.

**Condition query**

flatfoot OR "flat foot" OR "flat feet" OR "flexible flatfoot" OR "flexible pes planus" OR "pes planus" OR "pes planovalgus" OR planovalgus OR "low arch" OR overpronation OR "pronated foot"

**Intervention query**

exercise OR training OR strengthening OR rehabilitation OR "short foot" OR "foot exercise" OR "foot strengthening" OR "lower extremity exercise" OR "hip strengthening" OR "kinetic chain" OR "core stability" OR Pilates

The condition and intervention searches were merged and deduplicated to obtain the reported unique registry-record count.

**S7. Backward and forward citation searching**

**Sources:** Crossref REST API and OpenAlex API.

**Search date:** 18 July 2026.

**Seed reports:** 6.

**Backward searching:** 164 cited works were resolved from 173 unique reference identifiers.

**Forward searching:** 97 forward-citation links were retrieved.

Reference lists of eligible reports and relevant reviews were also checked, consistent with the supplementary-search procedure described in the main Methods.
