# Supplementary material for: Exercise programmes incorporating proximal kinetic-chain components in flexible flatfoot: a systematic review and meta-analysis of foot posture, neuromuscular, and dynamic outcomes
Source: Front Physiol. 2026 Oct 12;17:1959545. doi: 10.3389/fphys.2026.1959545 (PMC13696242; doi:10.3389/fphys.2026.1959545)
Supplement: Supplementary file 2 [file Table2.docx]

**Supplementary Material S2. Sensitivity analyses**

| **Outcome** | **Primary estimate (95% CI)** | **Sensitivity analysis** | **Sensitivity estimate (95% CI)** | **I²** | **Interpretation changed** |
| --- | --- | --- | --- | --- | --- |
| Navicular drop (k=11) | MD −2.34 mm (95% CI −3.30 to −1.37) | Change-score correlation r=0.30 | MD −2.28 mm (95% CI −3.05 to −1.51) | 75.5% | No |
| Navicular drop (k=11) | MD −2.34 mm (95% CI −3.30 to −1.37) | Change-score correlation r=0.70 | MD −2.25 mm (95% CI −3.01 to −1.48) | 85.7% | No |
| FPI-6 (k=4) | MD −1.67 points (95% CI −3.36 to 0.01) | Change-score correlation r=0.30 | MD -1.76 points (95% CI -3.50 to -0.02) | 80.0% | Yes |
| FPI-6 (k=4) | MD −1.67 points (95% CI −3.36 to 0.01) | Change-score correlation r=0.70 | MD -1.79 points (95% CI -3.50 to -0.09) | 89.0% | Yes |
| Plantar Arch Index (k=2) | MD −0.134 (95% CI −0.278 to 0.009) | Change-score correlation r=0.30 | MD −0.119 (95% CI −0.273 to 0.036) | 93.3% | No |
| Plantar Arch Index (k=2) | MD −0.134 (95% CI −0.278 to 0.009) | Change-score correlation r=0.70 | MD −0.119 (95% CI −0.273 to 0.035) | 96.6% | No |
| Y-Balance—anterior (k=2) | Hedges’ g=0.43 (95% CI −0.49 to 1.36) | Change-score correlation r=0.30 | Hedges’ g=0.37 (95% CI −0.17 to 0.91) | 0.0% | No |
| Y-Balance—anterior (k=2) | Hedges’ g=0.43 (95% CI −0.49 to 1.36) | Change-score correlation r=0.70 | Hedges’ g=0.55 (95% CI 0.01 to 1.10) | 0.0% | Yes |
| Y-Balance—posteromedial (k=2) | Hedges’ g=0.78 (95% CI −0.78 to 2.35) | Change-score correlation r=0.30 | Hedges’ g=0.63 (95% CI −0.62 to 1.88) | 79.9% | No |
| Y-Balance—posteromedial (k=2) | Hedges’ g=0.78 (95% CI −0.78 to 2.35) | Change-score correlation r=0.70 | Hedges’ g=0.93 (95% CI −0.94 to 2.80) | 89.9% | No |
| Y-Balance—posterolateral (k=2) | Hedges’ g=0.66 (95% CI −1.18 to 2.50) | Change-score correlation r=0.30 | Hedges’ g=0.43 (95% CI −1.27 to 2.12) | 88.9% | No |
| Y-Balance—posterolateral (k=2) | Hedges’ g=0.66 (95% CI −1.18 to 2.50) | Change-score correlation r=0.70 | Hedges’ g=0.64 (95% CI −1.91 to 3.18) | 94.4% | No |
| FPI-6 (k=3) | MD −1.67 points (95% CI −3.36 to 0.01) | Exclude Moghadasi | MD −1.58 points (95% CI −4.73 to 1.56) | 92.5% | No |
| Navicular drop (k=10) | MD −2.34 mm (95% CI −3.30 to −1.37) | Exclude Engkananuwat | MD −2.54 mm (95% CI −3.53 to −1.55) | 77.7% | No |
| Navicular drop (k=9) | MD −2.34 mm (95% CI −3.30 to −1.37) | Exclude Brijwasi and Alam | MD −1.99 mm (95% CI −2.95 to −1.04) | 80.2% | No |
| Y-Balance—anterior (k=1) | Hedges g 0.43 (95% CI −0.49 to 1.36) | Exclude Alam | Single-study g −0.04 (95% CI −0.80 to 0.73) | Not applicable | Not pooled |
| Y-Balance—posteromedial (k=1) | Hedges g 0.78 (95% CI −0.78 to 2.35) | Exclude Alam | Single-study g 0.00 (95% CI −0.77 to 0.77) | Not applicable | Not pooled |
| Y-Balance—posterolateral (k=1) | Hedges g 0.66 (95% CI −1.18 to 2.50) | Exclude Alam | Single-study g −0.27 (95% CI −1.04 to 0.51) | Not applicable | Not pooled |

Note. Analyses used random-effects REML models, with Knapp–Hartung 95% CIs for k≥3 and Wald CIs for exploratory k=2 analyses. No pooling was performed for k=1. Saleh dispersions were treated as SDs in primary and change-score FPI-6 analyses. Change-score correlations were r=0.30 or r=0.70. The original exclusions were added during revision and applied separately; the multicomponent exclusion removed Brijwasi and Alam. Moghadasi exclusion tested the influence of this study; Engkananuwat exclusion tested bilateral-data handling. I² uses the model-based definition. “Interpretation changed” refers only to whether the CI includes zero compared with the primary interval, not to clinical importance. k is the number of studies in the sensitivity analysis. MD, mean difference; FPI-6, six-item Foot Posture Index; CI, confidence interval.

Bilateral-data check. For Engkananuwat, 52 feet per group arose from 26 participants. The retained ICC=1 assumption gives DE=2 and n_eff=26 per group; published foot-level means and SDs are unchanged. The navicular-drop standard error was 0.25718 mm and its sampling variance was 0.06614 mm². The variance was entered once; no second design-effect multiplier was applied. The exclusion analysis removes this study rather than varying ICC and should not be interpreted as an ICC-parameter sensitivity analysis.

**Exploratory programme-matching analyses**

CM-only and CM+PM restrictions were compared with separate omissions of each represented category. REML and the primary interval rules were retained (Knapp–Hartung for k≥3; exploratory Wald intervals for k=2). For k=1, the study estimate and normal-approximation interval are shown, with no pooling, I² or τ². Identical retained samples are shown together.

**Table S2b. Navicular-drop programme-matching sensitivity analyses (MD, mm)**

| **Analysis** | **k/m/N** | **MD (95% CI)** | **I² (%)** | **τ²** | **Retained** | **Excluded** |
| --- | --- | --- | --- | --- | --- | --- |
| Primary | 11/11/410 | -2.34 (-3.30, -1.37) | 84.6 | 1.556 | ESE, RAG, ZAR, BRI, ENG, UTS, SAW, ALA, MUL, GG, GH | None |
| CM only | 5/5/172 | -1.88 (-3.09, -0.68) | 79.0 | 0.856 | ZAR, ENG, MUL, GG, GH | ESE, RAG, BRI, UTS, SAW, ALA |
| CM+PM / exclude UM | 9/9/333 | -1.99 (-2.95, -1.04) | 80.2 | 1.106 | ESE, RAG, ZAR, ENG, UTS, SAW, MUL, GG, GH | BRI, ALA |
| Exclude CM | 6/6/238 | -2.72 (-4.59, -0.85) | 85.3 | 2.469 | ESE, RAG, BRI, UTS, SAW, ALA | ZAR, ENG, MUL, GG, GH |
| Exclude PM | 7/7/249 | -2.44 (-3.71, -1.17) | 86.0 | 1.538 | ZAR, BRI, ENG, ALA, MUL, GG, GH | ESE, RAG, UTS, SAW |

All five rows use Knapp–Hartung intervals. Removing UM omits Brijwasi and Alam and reproduces the previously reported exclusion analysis. Primary and CM+PM models contain 12 and 10 original arm-to-comparator contrasts, respectively, reduced to 11 and 9 independent effects by combining the Raghav arms. Other rows have one original comparison per trial. In the CM-only navicular-drop model, the study weights were Zarali 2024, 18.48%; Engkananuwat, 26.61%; Mulchandani, 25.34%; Goo 2016a, 14.19%; and Goo 2016b, 15.37%. Engkananuwat and Mulchandani together contributed 52.0% (calculated from unrounded weights).

**Table S2c. Other outcomes and sparse programme-matching checks**

| **Outcome** | **Analysis** | **k/m/N** | **Effect (95% CI)** | **I² (%)** | **τ²** | **Retained** | **Excluded** |
| --- | --- | --- | --- | --- | --- | --- | --- |
| FPI-6 | Primary; no UM to omit | 4/4/157 | -1.67 (-3.36, 0.01) | 89.1 | 1.008 | MOG, ESE, RAG, SAL | None |
| FPI-6 | CM only / exclude PM | 1/1/22 | -2.00 (-2.80, -1.20) | NA | NA | MOG | ESE, RAG, SAL |
| FPI-6 | Exclude CM | 3/3/135 | -1.58 (-4.73, 1.56) | 92.5 | 1.478 | ESE, RAG, SAL | MOG |
| PAI | Primary; no UM to omit | 2/2/92 | -0.134 (-0.278, 0.009) | 96.5 | 0.010 | SAW, MUL | None |
| PAI | CM only / exclude PM | 1/1/52 | -0.208 (-0.250, -0.166) | NA | NA | MUL | SAW |
| PAI | Exclude CM | 1/1/40 | -0.061 (-0.095, -0.028) | NA | NA | SAW | MUL |
| YBT anterior | Primary; no PM to omit | 2/2/54 | 0.43 (-0.49, 1.36) | 65.1 | 0.291 | ZAR, ALA | None |
| YBT anterior | CM only / CM+PM / exclude UM | 1/1/26 | -0.04 (-0.80, 0.73) | NA | NA | ZAR | ALA |
| YBT anterior | Exclude CM | 1/1/28 | 0.91 (0.13, 1.69) | NA | NA | ALA | ZAR |
| YBT posteromedial | Primary; no PM to omit | 2/2/54 | 0.78 (-0.78, 2.35) | 86.5 | 1.102 | ZAR, ALA | None |
| YBT posteromedial | CM only / CM+PM / exclude UM | 1/1/26 | 0.00 (-0.77, 0.77) | NA | NA | ZAR | ALA |
| YBT posteromedial | Exclude CM | 1/1/28 | 1.59 (0.74, 2.45) | NA | NA | ALA | ZAR |
| YBT posterolateral | Primary; no PM to omit | 2/2/54 | 0.66 (-1.18, 2.50) | 90.1 | 1.584 | ZAR, ALA | None |
| YBT posterolateral | CM only / CM+PM / exclude UM | 1/1/26 | -0.27 (-1.04, 0.51) | NA | NA | ZAR | ALA |
| YBT posterolateral | Exclude CM | 1/1/28 | 1.61 (0.75, 2.47) | NA | NA | ALA | ZAR |

**Table S2d. Comparison-specific programme classifications**

| **Code** | **Trial / reports** | **Class** | **Selected comparison and classification basis** |
| --- | --- | --- | --- |
| MOG | Moghadasi 2025 | CM | CCE vs IFM+EFM: same local progression; added core/hip. Equal total exposure not established. |
| ALI | Ali 2025 | PM | Pilates+SFE vs SFE: common local programme; additional supervised Pilates and warm-up/cool-down. |
| ESE | Esen 2025 | PM | HEG vs CG: common SFE; extra supervised clam sessions. FEG not pooled. |
| RAG | Raghav 2024 | PM | GMax+SFE and GMed+SFE vs SFE: both PM; incomplete local-dose matching; two arms combined. |
| ZAR | Zarali 2023/2024 | CM | SFE+isometric hip abduction vs SFE: same local programme; CE arm not pooled. Two reports, one trial. |
| BRI | Brijwasi 2023 | UM | Comprehensive vs basic ankle exercise: extra SFE and gluteal work; 30 vs8 min. |
| ENG | Engkananuwat 2023 | CM | Same foot exercises with/without GMed: common local progression and follow-up; extra hip work. |
| UTS | Utsahachant 2023 | PM | SFLE vs SF: local task changes to wall squat/unilateral wall squat rather than standing SFE. |
| SAL | Saleh 2022 | PM | Backward walking+traditional PT vs traditional PT: additional supervised/dose exposure and local warm-up. |
| SAW | Sawant 2021 | PM | Proximal-to-distal+intrinsic vs intrinsic: calf-stretch allocation unclear; retained PM, not established matching. |
| ALA | Alam 2019 | UM | TP+iliopsoas training+towel curls vs towel curls: additional distinct local TP strengthening. |
| MUL | Mulchandani 2017 | CM | Gluteal+intrinsic vs intrinsic: same SFE/heel raise/toe curl prescription; total contact NR. |
| GG | Goo 2016a (GMax) | CM | GMax+toe spread vs toe spread: same100 toe-spread reps; extra3x20 GMax reps. |
| GH | Goo 2016b (hip external rotator) | CM | Hip external rotation+toe spread vs toe spread: same100 toe-spread reps; extra3x20 hip reps. |

CM, component-matched background local programme; PM, partial matching or incomplete local-dose/delivery reporting; UM, added distinct local treatment components; NR, not reported.
