# Supplementary material for: Exercise programmes incorporating proximal kinetic-chain components in flexible flatfoot: a systematic review and meta-analysis of foot posture, neuromuscular, and dynamic outcomes
Source: Front Physiol. 2026 Oct 12;17:1959545. doi: 10.3389/fphys.2026.1959545 (PMC13696242; doi:10.3389/fphys.2026.1959545)
Supplement: Supplementary file 3 [file Table3.docx]

**Supplementary Material S3 Individual trial outcome estimates**

**Proprioception**

| **Study and comparison** | **Outcome and unit** | **Time and n** | **Between-group estimate or descriptive result** |
| --- | --- | --- | --- |
| Zarali 2023 [33] | Joint-position sense error (dominant limb), degrees | 6 weeks n I/C 13/13 | MD 0.23 (95% CI −1.12 to 1.58) |

**Strength**

| **Study and comparison** | **Outcome and unit** | **Time and n** | **Between-group estimate or descriptive result** |
| --- | --- | --- | --- |
| Engkananuwat 2023 [16] | Flexor hallucis brevis strength (bilateral measurements), N | 8 weeks n I/C 26/26 | MD −0.09 (95% CI −2.89 to 2.71) |
| Engkananuwat 2023 [16] | Gluteus medius strength (bilateral measurements), N | 8 weeks n I/C 26/26 | MD 12.81 (95% CI 5.76 to 19.86) |
| Engkananuwat 2023 [16] | Tibialis posterior strength (bilateral measurements), N | 8 weeks n I/C 26/26 | MD −2.01 (95% CI −7.20 to 3.18) |
| Zarali 2023 [33] | Dorsiflexion strength (dominant limb), kg/kg body weight | 6 weeks n I/C 13/13 | MD −0.36 (95% CI −1.71 to 0.99) |
| Zarali 2023 [33] | Plantar-flexion strength (dominant limb), kg/kg body weight | 6 weeks n I/C 13/13 | MD −1.50 (95% CI −4.18 to 1.18) |

**Motor control**

| **Study and comparison** | **Outcome and unit** | **Time and n** | **Between-group estimate or descriptive result** |
| --- | --- | --- | --- |
| Utsahachant 2023 [38] | Intrinsic foot muscle test (selected foot; clinical ordinal test), ordinal score | 6 weeks n I/C 15/15 | I 3.00 [IQR 3.00–3.00]; C 3.00 [2.00–3.00] |

**Muscle activity**

| **Study and comparison** | **Outcome and unit** | **Time and n** | **Between-group estimate or descriptive result** |
| --- | --- | --- | --- |
| Alam 2019 [32] | Abductor hallucis EMG (dominant; treadmill walking), %MVIC | 6 weeks n I/C 14/14 | MD 143.90 (95% CI 68.51 to 219.29) |
| Alam 2019 [32] | Tibialis anterior EMG (dominant; treadmill walking), %MVIC | 6 weeks n I/C 14/14 | MD −547.30 (95% CI −717.85 to −376.75) |
| Goo 2016a [41] | AbdH EMG (selected/unclear; foot flat), %MVC | 4 weeks n I/C 9/9 | MD 0.00 (95% CI −7.92 to 7.92) |
| Goo 2016a [41] | AbdH EMG (selected/unclear; heel strike), %MVC | 4 weeks n I/C 9/9 | MD −3.50 (95% CI −10.84 to 3.84) |
| Goo 2016a [41] | AbdH EMG (selected/unclear; toe off), %MVC | 4 weeks n I/C 9/9 | MD 2.00 (95% CI −8.04 to 12.04) |
| Goo 2016a [41] | GM EMG (selected/unclear; foot flat), %MVC | 4 weeks n I/C 9/9 | MD 5.40 (95% CI −2.29 to 13.09) |
| Goo 2016a [41] | GM EMG (selected/unclear; heel strike), %MVC | 4 weeks n I/C 9/9 | MD 11.30 (95% CI −0.74 to 23.34) |
| Goo 2016a [41] | GM EMG (selected/unclear; toe off), %MVC | 4 weeks n I/C 9/9 | MD −2.30 (95% CI −12.64 to 8.04) |
| Goo 2016a [41] | TA EMG (selected/unclear; foot flat), %MVC | 4 weeks n I/C 9/9 | MD 0.70 (95% CI −8.60 to 10.00) |
| Goo 2016a [41] | TA EMG (selected/unclear; heel strike), %MVC | 4 weeks n I/C 9/9 | MD 1.00 (95% CI −7.61 to 9.61) |
| Goo 2016a [41] | TA EMG (selected/unclear; toe off), %MVC | 4 weeks n I/C 9/9 | MD 4.20 (95% CI 0.25 to 8.15) |
| Goo 2016a [41] | VMO EMG (selected/unclear; foot flat), %MVC | 4 weeks n I/C 9/9 | MD 6.30 (95% CI 0.51 to 12.09) |
| Goo 2016a [41] | VMO EMG (selected/unclear; heel strike), %MVC | 4 weeks n I/C 9/9 | MD 14.10 (95% CI 6.13 to 22.07) |
| Goo 2016a [41] | VMO EMG (selected/unclear; toe off), %MVC | 4 weeks n I/C 9/9 | MD −3.50 (95% CI −13.45 to 6.45) |
| Goo 2016b [42] | AbdH EMG (selected/unclear; stair down), %MVC | 4 weeks n I/C 10/10 | MD 3.00 (95% CI −5.46 to 11.46) |
| Goo 2016b [42] | AbdH EMG (selected/unclear; stair up), %MVC | 4 weeks n I/C 10/10 | MD 0.88 (95% CI −8.10 to 9.86) |
| Goo 2016b [42] | GM EMG (selected/unclear; stair down), %MVC | 4 weeks n I/C 10/10 | MD −10.30 (95% CI −18.84 to −1.76) |
| Goo 2016b [42] | GM EMG (selected/unclear; stair up), %MVC | 4 weeks n I/C 10/10 | MD −1.40 (95% CI −9.92 to 7.12) |
| Goo 2016b [42] | TA EMG (selected/unclear; stair down), %MVC | 4 weeks n I/C 10/10 | MD −0.20 (95% CI −11.61 to 11.21) |
| Goo 2016b [42] | TA EMG (selected/unclear; stair up), %MVC | 4 weeks n I/C 10/10 | MD 3.30 (95% CI −4.66 to 11.26) |
| Goo 2016b [42] | VMO EMG (selected/unclear; stair down), %MVC | 4 weeks n I/C 10/10 | MD −8.70 (95% CI −16.77 to −0.63) |
| Goo 2016b [42] | VMO EMG (selected/unclear; stair up), %MVC | 4 weeks n I/C 10/10 | MD −12.00 (95% CI −20.74 to −3.26) |

**Plantar pressure**

| **Study and comparison** | **Outcome and unit** | **Time and n** | **Between-group estimate or descriptive result** |
| --- | --- | --- | --- |
| Zarali 2024 [15] | Maximum pressure (dominant foot; pedoscan/one-leg stance), N | 6 weeks n I/C 15/15 | MD −0.10 (95% CI −1.47 to 1.27) |
| Engkananuwat 2023 [16] | Lateral forefoot pressure (bilateral measurements), kPa | 8 weeks n I/C 26/26 | MD 1.68 (95% CI −6.25 to 9.61) |
| Engkananuwat 2023 [16] | Lateral hindfoot pressure (bilateral measurements), kPa | 8 weeks n I/C 26/26 | MD 1.90 (95% CI −11.35 to 15.15) |
| Engkananuwat 2023 [16] | Medial forefoot pressure (bilateral measurements), kPa | 8 weeks n I/C 26/26 | MD −8.61 (95% CI −13.42 to −3.80) |
| Engkananuwat 2023 [16] | Medial hindfoot pressure (bilateral measurements), kPa | 8 weeks n I/C 26/26 | MD −9.85 (95% CI −21.09 to 1.39) |
| Engkananuwat 2023 [16] | Midfoot pressure (bilateral measurements), kPa | 8 weeks n I/C 26/26 | MD 0.69 (95% CI −4.42 to 5.80) |

**Gait kinematics**

| **Study and comparison** | **Outcome and unit** | **Time and n** | **Between-group estimate or descriptive result** |
| --- | --- | --- | --- |
| Utsahachant 2023 [38] | Largest MLA angle during loading response (selected foot; gait/NDT), degrees | 6 weeks n I/C 15/15 | MD −0.62 (95% CI −5.64 to 4.40) |
| Utsahachant 2023 [38] | Largest MLA angle during push-off (selected foot; gait/NDT), degrees | 6 weeks n I/C 15/15 | MD 0.50 (95% CI −5.26 to 6.26) |
| Utsahachant 2023 [38] | MLA deformation angle (selected foot; gait/NDT), degrees | 6 weeks n I/C 15/15 | MD 0.94 (95% CI −1.99 to 3.87) |
| Utsahachant 2023 [38] | Midfoot eversion excursion (selected foot; gait/NDT), degrees | 6 weeks n I/C 15/15 | MD 0.32 (95% CI −0.88 to 1.52) |
| Utsahachant 2023 [38] | Smallest MLA angle during midstance (selected foot; gait), degrees | 6 weeks n I/C 15/15 | I 149.15 [IQR 145.98–153.37]; C 147.67 [143.57–154.39] |
| Utsahachant 2023 [38] | Time to lowest MLA (selected foot; gait/NDT), % stance | 6 weeks n I/C 15/15 | MD −1.40 (95% CI −3.43 to 0.63) |

**Dynamic foot function**

| **Study and comparison** | **Outcome and unit** | **Time and n** | **Between-group estimate or descriptive result** |
| --- | --- | --- | --- |
| Utsahachant 2023 [38] | CPEI (selected foot; gait/NDT), % | 6 weeks n I/C 15/15 | MD 0.84 (95% CI −2.13 to 3.81) |

**Static foot posture**

| **Study and comparison** | **Outcome and unit** | **Time and n** | **Between-group estimate or descriptive result** |
| --- | --- | --- | --- |
| Brijwasi 2023 [17] | Medial longitudinal arch angle (selected foot; standing arch angle), degrees | 6 weeks n I/C 25/24 | MD 20.00 (95% CI 15.47 to 24.53) |
| Engkananuwat 2023 [16] | Arch height index (bilateral measurements), ratio | 8 weeks n I/C 26/26 | MD 0.010 (95% CI 0.006 to 0.014) |

**Footprint**

| **Study and comparison** | **Outcome and unit** | **Time and n** | **Between-group estimate or descriptive result** |
| --- | --- | --- | --- |
| Esen 2025 [34] | Clarke angle (dominant foot; clinical/footprint), degrees | 6 weeks n I/C 18/19 | I 30.49 [IQR 29.10–30.87]; C 30.35 [29.58–30.63] |
| Esen 2025 [34] | Staheli index (dominant foot; clinical/footprint), ratio | 6 weeks n I/C 18/19 | I 0.59 [IQR 0.58–0.61]; C 0.60 [0.59–0.61] |
| Esen 2025 [34] | Wejflog index (dominant foot; clinical/footprint), score | 6 weeks n I/C 18/19 | I 2.79 [IQR 2.68–2.89]; C 2.82 [2.70–2.97] |
| Zarali 2024 [15] | Foot rotation (dominant foot; pedoscan/one-leg stance), degrees | 6 weeks n I/C 15/15 | MD 0.45 (95% CI −0.10 to 1.00) |
| Zarali 2024 [15] | Foot surface area (dominant foot; pedoscan/one-leg stance), cm2 | 6 weeks n I/C 15/15 | MD −2.00 (95% CI −10.35 to 6.35) |

**Toe alignment**

| **Study and comparison** | **Outcome and unit** | **Time and n** | **Between-group estimate or descriptive result** |
| --- | --- | --- | --- |
| Esen 2025 [34] | Fifth-toe varus angle (dominant foot; footprint photograph), degrees | 6 weeks n I/C 18/19 | MD 0.55 (95% CI −0.70 to 1.80) |
| Esen 2025 [34] | Hallux valgus angle (dominant foot; footprint photograph), degrees | 6 weeks n I/C 18/19 | MD −0.09 (95% CI −2.45 to 2.27) |

**Static balance**

| **Study and comparison** | **Outcome and unit** | **Time and n** | **Between-group estimate or descriptive result** |
| --- | --- | --- | --- |
| Raghav 2024 [37] GMax + SFE vs SFE | Stork stance left (left; clinical balance/foot posture), seconds | 4 weeks n I/C 18/18 | MD 2.86 (95% CI 1.17 to 4.55) |
| Raghav 2024 [37] GMed + SFE vs SFE | Stork stance left (left; clinical balance/foot posture), seconds | 4 weeks n I/C 18/18 | MD 6.16 (95% CI 4.48 to 7.84) |
| Raghav 2024 [37] GMax + SFE vs SFE | Stork stance right (right; clinical balance/foot posture), seconds | 4 weeks n I/C 18/18 | MD 2.66 (95% CI 1.27 to 4.05) |
| Raghav 2024 [37] GMed + SFE vs SFE | Stork stance right (right; clinical balance/foot posture), seconds | 4 weeks n I/C 18/18 | MD 5.11 (95% CI 3.71 to 6.51) |
| Zarali 2024 [15] | AP sway (dominant foot; pedoscan/one-leg stance), mm | 6 weeks n I/C 15/15 | MD −17.42 (95% CI −44.96 to 10.12) |
| Zarali 2024 [15] | Average sway speed (dominant foot; pedoscan/one-leg stance), mm/s | 6 weeks n I/C 15/15 | MD −1.84 (95% CI −4.70 to 1.02) |
| Zarali 2024 [15] | CoP trace length (dominant foot; pedoscan/one-leg stance), mm | 6 weeks n I/C 15/15 | MD −15.85 (95% CI −43.93 to 12.23) |
| Zarali 2024 [15] | ML sway (dominant foot; pedoscan/one-leg stance), mm | 6 weeks n I/C 15/15 | MD −17.87 (95% CI −34.80 to −0.94) |
| Zarali 2024 [15] | Sway area (dominant foot; pedoscan/one-leg stance), mm2 | 6 weeks n I/C 15/15 | MD −8.08 (95% CI −61.06 to 44.90) |
| Engkananuwat 2023 [16] | AP displacement (bilateral measurements), cm | 8 weeks n I/C 26/26 | MD −0.49 (95% CI −0.90 to −0.08) |
| Engkananuwat 2023 [16] | ML displacement (bilateral measurements), cm | 8 weeks n I/C 26/26 | MD −0.45 (95% CI −0.72 to −0.18) |

**Dynamic balance**

| **Study and comparison** | **Outcome and unit** | **Time and n** | **Between-group estimate or descriptive result** |
| --- | --- | --- | --- |
| Ali 2025 [36] | Back average deviation (participant; Biodex/FFI), score | 5 weeks n I/C 22/22 | MD −1.01 (95% CI −1.33 to −0.69) |
| Ali 2025 [36] | Front average deviation (participant; Biodex/FFI), score | 5 weeks n I/C 22/22 | MD −1.00 (95% CI −1.30 to −0.70) |
| Ali 2025 [36] | Left average deviation (participant; Biodex/FFI), score | 5 weeks n I/C 22/22 | MD −0.71 (95% CI −0.93 to −0.49) |
| Ali 2025 [36] | Right average deviation (participant; Biodex/FFI), score | 5 weeks n I/C 22/22 | MD −0.93 (95% CI −1.23 to −0.63) |
| Raghav 2024 [37] GMax + SFE vs SFE | mSEBT left composite/sum (left; clinical balance/foot posture), score/cm sum | 4 weeks n I/C 18/18 | MD 20.98 (95% CI 8.53 to 33.43) |
| Raghav 2024 [37] GMed + SFE vs SFE | mSEBT left composite/sum (left; clinical balance/foot posture), score/cm sum | 4 weeks n I/C 18/18 | MD 29.41 (95% CI 18.30 to 40.52) |
| Raghav 2024 [37] GMax + SFE vs SFE | mSEBT right composite/sum (right; clinical balance/foot posture), score/cm sum | 4 weeks n I/C 18/18 | MD 14.03 (95% CI 1.05 to 27.01) |
| Raghav 2024 [37] GMed + SFE vs SFE | mSEBT right composite/sum (right; clinical balance/foot posture), score/cm sum | 4 weeks n I/C 18/18 | MD 18.03 (95% CI 9.01 to 27.05) |
| Engkananuwat 2023 [16] | Anterior reach (bilateral measurements), % limb length | 8 weeks n I/C 26/26 | MD 1.43 (95% CI −1.71 to 4.57) |
| Engkananuwat 2023 [16] | Lateral reach (bilateral measurements), % limb length | 8 weeks n I/C 26/26 | MD −0.88 (95% CI −5.82 to 4.06) |
| Engkananuwat 2023 [16] | Medial reach (bilateral measurements), % limb length | 8 weeks n I/C 26/26 | MD 1.10 (95% CI −3.36 to 5.56) |
| Engkananuwat 2023 [16] | Posterior reach (bilateral measurements), % limb length | 8 weeks n I/C 26/26 | MD −0.62 (95% CI −4.74 to 3.50) |
| Saleh 2022 [35] | Limits of Stability (participant; FPI/Biodex limits), % | 6 weeks n I/C 22/22 | MD 7.40 (95% CI 3.75 to 11.05) |

**Patient-reported function**

| **Study and comparison** | **Outcome and unit** | **Time and n** | **Between-group estimate or descriptive result** |
| --- | --- | --- | --- |
| Ali 2025 [36] | Foot Function Index (participant; Biodex/FFI), % | 5 weeks n I/C 22/22 | MD −8.55 (95% CI −11.47 to −5.63) |

**Adherence**

| **Study and comparison** | **Outcome and unit** | **Time and n** | **Between-group estimate or descriptive result** |
| --- | --- | --- | --- |
| Ali 2025 [36] | Home-exercise compliance | 5 weeks | 85.7% vs 88.6% complied with home exercise |
| Esen 2025 [34] | Home short-foot-exercise compliance | 6 weeks | 95% vs 95% |
| Brijwasi 2023 [17] | Exercise compliance | 6 weeks | Compliance was not assessed |
| Engkananuwat 2023 [16] | Days of home exercise completed | 8 weeks | 35.7 of 40 prescribed days vs 36.2 of 40 days |

**Withdrawals/adverse events**

| **Study and comparison** | **Outcome and unit** | **Time and n** | **Between-group estimate or descriptive result** |
| --- | --- | --- | --- |
| Esen 2025 [34] | Withdrawals and reasons across three randomised groups | 6 weeks | Three withdrawals due to discomfort were reported across the randomised groups; group-specific safety denominators were not reported. |
| Brijwasi 2023 [17] | Reported adverse events among completers | 6 weeks | No adverse events were reported among participants who completed follow-up; three participants were lost to follow-up |

Notes. MD, mean difference; CI, confidence interval; I/C, intervention/comparator; SFE, short-foot exercise; GMax/GMed, gluteus maximus/medius; AbdH, abductor hallucis; GM, gluteus maximus; TA, tibialis anterior; VMO, vastus medialis; EMG, electromyography; MVC/MVIC, maximal (voluntary isometric) contraction; MLA, medial longitudinal arch; AP/ML, anteroposterior/mediolateral; CPEI, centre-of-pressure excursion index; mSEBT, modified Star Excursion Balance Test.
