# Supplementary material for: Exercise programmes incorporating proximal kinetic-chain components in flexible flatfoot: a systematic review and meta-analysis of foot posture, neuromuscular, and dynamic outcomes
Source: Front Physiol. 2026 Oct 12;17:1959545. doi: 10.3389/fphys.2026.1959545 (PMC13696242; doi:10.3389/fphys.2026.1959545)
Supplement: Supplementary file 4 [file Table4.docx]

**Supplementary Material S4**

**Outcome specific GRADE rationales**

This supplement explains the domain judgements accompanying Table 3. Certainty concerns intervention-end differences between whole exercise programmes and local or basic foot–ankle exercise comparators in the eligible study-defined populations. Navicular drop was rated low; FPI-6, exploratory Plantar Arch Index, and all three exploratory Y-Balance directions were rated very low. Reference numbers correspond to the main manuscript.

**Assessment scope**

Randomised evidence started at high certainty. Downgrading by one or two levels indicates serious or very serious limitations, respectively; the lowest category is very low. These judgements apply to the primary final-value estimates. Sensitivity analyses inform their interpretation but do not replace them. Risk of bias concerns limitations within studies, inconsistency concerns unexplained differences between studies, and imprecision concerns uncertainty around the pooled mean. A shared concern was not automatically counted in more than one domain.

**Indirectness and publication bias**

Indirectness was not downgraded for the defined question: the studies directly compared eligible exercise programmes and measured the specified posture or reach outcomes at the end of treatment. Two trials explicitly reported flexibility testing; the other 12 reported posture or navicular-drop criteria without separately reporting a flexibility-confirmation test. This reporting gap limits verification of a common flexible-flatfoot definition across samples; it does not establish which actual phenotypes were enrolled in those 12 trials. This judgement does not support extrapolation to exclusively flexibility-confirmed symptomatic patients, older clinical populations, isolated proximal-component effects, or long-term benefit. Static posture and reach distance were assessed as outcomes in their own right, not as substitutes for pain relief, participation, or injury prevention.

No additional publication-bias downgrade was applied. The navicular-drop funnel plot and Egger test (p=0.286) did not establish absence of bias, particularly given heterogeneity. The other pools contained too few studies for informative formal asymmetry testing. Four unavailable reports remained a potential source of availability bias of unknown direction; their effects and eligibility could not be established. Small study numbers were considered under imprecision rather than automatically penalised again as publication bias.

**Navicular drop**

Eleven trials involving 410 participants yielded MD −2.34 mm (95% CI −3.30 to −1.37; I²=84.6%). Risk of bias was serious (−1): concerns in allocation, assessor-dependent measurement, and selection of reported results affected a substantial part of the evidence. These concerns have a plausible pathway to changing measured between-group differences, without establishing their direction. Inconsistency was serious (−1): individual MDs ranged from approximately −0.36 to −5.00 mm, and dispersion remained substantial after the completed exclusions. Differences in programme content and measurement may contribute, but were not established effect modifiers. Predominantly concordant direction supported one rather than two levels of downgrading for inconsistency. In the exploratory analyses added during revision, navicular-drop heterogeneity remained substantial both after excluding UM comparisons (I²=80.2%) and within CM comparisons alone (I²=79.0%; Supplementary Material S2, Table S2b). Matching background local content therefore did not resolve inconsistency or establish equal total exercise exposure; the low-certainty rating was retained.

Imprecision was not downgraded for the limited judgement that the average short-term navicular-drop value was lower: the primary interval and completed exclusion estimates retained that direction. This is not a judgement that the difference is clinically important. No validated patient-anchored threshold or prespecified optimal information size was used. Indirectness and publication bias were assessed as described above. Overall certainty was low.

**Foot Posture Index**

Four trials involving 157 participants yielded MD −1.67 points (95% CI −3.36 to 0.01; I²=89.1%). Risk of bias was serious (−1) because allocation, observational scoring, and reporting concerns affected influential studies. Inconsistency was serious (−1): point estimates were directed towards lower scores but differed markedly in magnitude, and heterogeneity remained high after excluding one influential study. The downgrade was not based on I² alone or on differences between final-value and change-score analyses.

Imprecision was serious (−1): the primary interval allowed appreciable score lowering as well as little or no difference, with only four contributing trials. Change-score intervals narrowly excluded zero, while an exclusion analysis admitted opposite directions; these findings reinforce uncertainty without replacing the primary interval. The main interval did not allow comparably large differences in both directions, supporting one rather than two levels of downgrading. No individual MDC was treated as a patient-important threshold. Indirectness and publication bias were assessed as above. Overall certainty was very low.

**Exploratory Plantar Arch Index**

Two trials involving 92 participants yielded MD −0.134 (95% CI −0.278 to 0.009; I²=96.5%). Risk of bias was serious (−1): both Sawant and Mulchandani [39, 40] had concerns in the randomisation, outcome-measurement, and reported-result domains. Allocation concealment was insufficiently described, and footprint acquisition and width measurements involved assessor-dependent procedures. Limited reporting was not interpreted as proof that blinding or randomisation had failed, but these concerns applied to the whole evidence body.

Inconsistency was serious (−1): the individual MDs were approximately −0.061 and −0.208, with non-overlapping intervals. The consistent direction but markedly different magnitude supported one level of downgrading. Imprecision was serious (−1): the pooled interval allowed appreciable ratio lowering and little or no difference, with sparse information and no validated clinical threshold. This judgement concerns uncertainty in the pooled mean, separately from between-study dispersion. Indirectness referred specifically to this footprint ratio, not another arch-index definition or functional benefit. Publication bias was assessed as above. Overall certainty was very low.

**Exploratory Y Balance Test directions**

Each direction included the same two trials and 54 participants. Hedges g was 0.43 (95% CI −0.49 to 1.36) anteriorly, 0.78 (−0.78 to 2.35) posteromedially, and 0.66 (−1.18 to 2.50) posterolaterally. Risk of bias was serious (−1) in each direction because concerns about deviations, missingness, assessor-dependent measurement, or result selection affected the contributing evidence. Inconsistency was serious (−1): one trial reported estimates near zero, whereas the other reported larger positive estimates. The source of this divergence could not be reliably separated with two trials.

Imprecision was serious (−1) for anterior reach and very serious (−2) for posteromedial and posterolateral reach. All intervals allowed different directions of performance change; the latter two allowed especially large differences in both directions. These decisions considered the interval width and sparse information together, not the study count or a p-value cut-off alone. Conventional standardised-effect thresholds were not treated as validated MCIDs. Indirectness concerned reach performance rather than gait, falls, or injury. Publication bias was assessed as above. All three directions had very-low-certainty evidence and were not treated as independent replication of one another.
