# Supplementary material for: Exercise programmes incorporating proximal kinetic-chain components in flexible flatfoot: a systematic review and meta-analysis of foot posture, neuromuscular, and dynamic outcomes
Source: Front Physiol. 2026 Oct 12;17:1959545. doi: 10.3389/fphys.2026.1959545 (PMC13696242; doi:10.3389/fphys.2026.1959545)
Supplement: Supplementary file 5 [file Table5.docx]

**Supplementary Material S5. Report availability and retrieval**

Four reports could not be retrieved and were classified as “Reports not retrieved” in the PRISMA flow diagram. The available bibliographic records or abstracts did not provide sufficient information to establish eligibility. Full reports were sought through bibliographic and title searches; author contact and interlibrary loan were not undertaken.

**1. Andreou and Triantafyllides (2004)**

Flexible pes planovalgus. Early physiotherapy.

The available conference abstract describes 45 children aged 3–6 years and compares physiotherapy with no physiotherapy. Randomisation could not be established. The complete report was not retrieved.

Record: https://www.cev.org.br/biblioteca/flexible-pes-planovalgus-early-physiotherapy/

**2. Shiian (1986)**

Therapeutic physical exercise and massage in scoliosis and flatfoot in schoolchildren. PMID: 3643370.

The title refers to exercise and massage for schoolchildren with scoliosis and/or flatfoot. Allocation, comparison groups, and eligibility could not be established. The complete report was not retrieved.

Record: https://pubmed.ncbi.nlm.nih.gov/3643370/

**3. Kubícek and Kubícková (1973)**

Rehabilitation of children—convalescence using corrective positions and exercises. Prevention of spinal diseases and flatfoot. PMID: 4700973.

The title refers to corrective positioning and exercise in children. Randomisation and comparison groups could not be established. The PubMed record contains no abstract, and the complete report was not retrieved.

Record: https://pubmed.ncbi.nlm.nih.gov/4700973/

**4. Brandt (1950)**

Funktionstreaning ved platfodethed [Functional training in flatfoot]. Ugeskr Laeger. 1950;112:1018–1019. PMID: 14782246.

The title refers to functional training for flatfoot. Participant age, comparison groups, and study design could not be established. The PubMed record contains no abstract, and the complete report was not retrieved.

Record: https://pubmed.ncbi.nlm.nih.gov/14782246/

**Disposition of three other reports**

Erten et al. (2025; DOI: 10.1089/g4h.2024.0179) was excluded because the intervention did not meet the eligibility criteria. Mahmoud et al. (2022; DOI: 10.3233/IES-210178) was excluded because non-exercise co-interventions differed between groups: the traditional physical therapy programme included lumbar transcutaneous electrical nerve stimulation and massage in addition to exercise (Methods, sections 2.6.1–2.6.3, pp. 148–149). Exercise in foot disabilities (1984) was excluded because the intervention did not meet the eligibility criteria. These three reports were classified as eligibility exclusions.
